# Fluoroether Design Enables High‐Voltage All‐Solid‐State Lithium Metal Batteries

**DOI:** 10.1002/adma.202506020

**Published:** 2025-07-01

**Authors:** Yong Chen, Xu Yang, Tianyi Wang, Xiao Tang, Dongfang Li, Shijian Wang, Yaojie Lei, Yu Han, Shimou Chen, Michel Armand, Doron Aurbach, Guoxiu Wang

**Affiliations:** ^1^ Centre for Clean Energy Technology University of Technology Sydney Broadway Sydney NSW 2007 Australia; ^2^ School of Chemistry and Chemical Engineering Qingdao University Qingdao Shandong 266071 P.R. China; ^3^ State Key Laboratory of Chemical Resource Engineering Beijing Key Laboratory of Electrochemical Process and Technology of Materials Beijing University of Chemical Technology Beijing 100029 P.R. China; ^4^ Centre for Cooperative Research on Alternative Energies (CIC EnergiGUNE) Basque Research and Technology Alliance (BRTA) Alava Technology Park Albert Einstein 48 Vitoria‐Gasteiz 01510 Spain; ^5^ Department of Chemistry BINA‐Bar‐Ilan Institute of Nanotechnology and Advanced Materials and INIES ‐Israel National Institute for Energy Storage Bar‐Ilan University Ramat‐Gan 5290002 Israel

**Keywords:** all‐solid‐state lithium‐metal batteries, composite polymer electrolyte, high durability, high voltage

## Abstract

Developing high‐voltage all‐solid‐state lithium metal batteries (ASSLMBs) holds transformative potential for next‐generation energy storage technologies but remains a formidable challenge. Herein, a new prototype design is presented that integrates fluorinated ether segments into the traditional oxide nanocomposite phase, enabling poly(ethylene oxide)‐based composite electrolytes with exceptional anti‐oxidation durability and enhance overall electrochemical performance. Through a combination of experimental and computational analyses, it is demonstrated that the superior performance is attributed to the formation of reconstructed Li⁺ solvation with weakly coordinating environments. The proposed formulation exhibits excellent Li‐metal compatibility, enabling stable cycling in symmetric Li||Li cells for over 9500 h. The solid‐state electrolyte also exhibits outstanding high‐voltage stability with LiNi_0.8_Co_0.1_Mn_0.1_O_2_ cathodes, extending the operational voltage from 4.0 to 4.5 V. Moreover, the LiMn_1‐x_Fe_x_PO_4_||Li cells have delivered remarkable cycling performance, achieving over 1200 cycles with 99% capacity retention after 500 cycles. This work establishes an innovative platform for designing electrolytes with superior antioxidation properties and enhance structural durability, paving the way for the advancement of high‐voltage all‐solid‐state lithium metal batteries.

## Graphical abstract

A durable high‐voltage all‐solid‐state electrolyte is developed by incorporating a novel organic/inorganic hybrid porous component with a fluoroether design. Due to the weakened Li^+^ coordination structure, the proposed high‐voltage SSE exhibits durable antioxidation. The optimized electrolytes show ultra‐stable cycling performance in Li||Li cells, LiNi_0.8_Co_0.1_Mn_0.1_O_2_||Li cells, and LiMn_0.6_Fe_0.4_PO_4_||Li cells even under 4.5V.

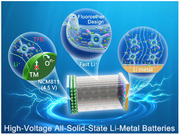

## 1 Introduction

All‐solid‐state lithium metal batteries (ASSLMBs) are widely recognized as promising next‐generation batteries, offering high energy density and enhanced safety.^[^ ^1^ ^]^ The key innovation lies in replacing flammable and volatile liquid electrolytes with robust solid‐state electrolytes (SSEs), which effectively eliminate safety concerns and premature battery failure.^[^ ^2^ ^]^ Among the advanced SSEs developed to date, solid polymer electrolytes stand out for their scalability in manufacturing, low interfacial resistance, and superior flexibility.^[^ ^2^, ^3^ ^]^ Poly(ethylene oxide) (PEO)‐based SSEs have garnered extensive attention owing to their well‐balanced properties, including a low glass transition temperature, excellent capacity for dissolving Li salts, and good compatibility with lithium metal anodes.^[^ ^4^ ^]^ Therefore, integrating high‐voltage cathodes with PEO‐based SSEs holds promise for harnessing the high specific capacity of lithium metal anodes (3860 mAh g⁻¹).^[^ ^5^ ^]^ However, despite extensive research, PEO‐based SSEs still encounter significant challenges, such as decomposition at high voltage and low ionic conductivity, which impede their practical application, particularly in high‐voltage ASSLMBs.^[^ ^1^, ^6^ ^]^

For high‐voltage ASSLMBs, the primary challenge encountered by PEO‐based SSEs lies in their intrinsic incompatibility with high‐voltage cathodes. It is widely acknowledged that the poor electrochemical performance is primarily due to the electrochemical/physicochemical instability of their components, including PEO matrices and cathode materials. The oxidative decomposition potential of the ether‐oxygen units containing (−C−O−C−) in PEO polymers is below 4V,^[^ ^7^, ^8^ ^]^ with hydroxyl (−OH) end groups serving as primary initiators of electrochemical degradation.^[^ ^9^, ^10^ ^]^ In addition to possible cathodes’ structural collapse, the electrolyte degradation on high capacity/high voltage Ni‐rich LiNi_1‐x‐y_Co_x_O_2_ (NCM) cathodes is intensified by LiOH and Li_2_CO_3_ residues on the cathodes’ surface, as well as the formation of high‐valence transition metal cations during charging (Ni^4+^).^[^ ^9^ ^]^ Furthermore, it was found that PEO‐based SSEs in high‐voltage batteries exhibit erratic voltage fluctuations during the initial cycling, which frequently results in battery failure.^[^ ^11^ ^]^ This failure primarily originates from excessive lithium dendrite growth on the Li anodes, rather than electrolyte decomposition at the high‐voltage cathode.^[^ ^12^ ^]^ Moreover, the “cross‐talking” effect between electrodes, induced by Li salt decomposition and transition metal dissolution, represents a crucial influence on deleterious reactions.^[^ ^13^, ^14^, ^15^ ^]^

Various strategies have been proposed to address these issues in high‐voltage ASSLMBs, such as incorporating inorganic fillers, modifying the PEO matrix,^[^ ^16^, ^17^ ^]^ developing novel salts,^[^ ^18^ ^]^ and constructing stable interfaces.^[^ ^9^, ^19^ ^]^ One of the pivotal strategies involves the engineering of composite electrolytes, wherein the incorporation of inorganic inert fillers or ion‐conducting ceramic fillers emerges as a promising approach to augment the high‐voltage stability of PEO‐based SSEs. These enhancements are primarily attributed to several crucial mechanisms, including Lewis acid‐base interactions and oxygen vacancy effects.^[^ ^20^, ^21^, ^22^ ^]^ Unfortunately, despite the inclusion of high‐cost superionic conductors, the improvement in electrochemical performance remained limited due to the considerable interfacial resistance between organic and inorganic components, as well as the complex high‐voltage failure mechanisms inherent to PEO‐based SSEs. Moreover, the incompatibility between organic and inorganic materials amplifies the risk of nanoparticle re‐agglomeration. This limits the seamless transition between “ceramic‐in‐polymer” and “polymer‐in‐ceramic” architectures, particularly at elevated temperatures.^[^ ^23^, ^24^ ^]^

Although organic grafting on inorganic fillers enhances film formation and nanocomposite phase dispersion, it does not inherently improve ionic transport efficiency at the filler‐electrolyte interface.^[^ ^25^, ^26^ ^]^ The observed increase in ionic conductivity is largely attributed to the strong coordination effects of residual solvents, which significantly compromise high‐temperature stability.^[^ ^27^, ^28^, ^29^ ^]^ Another effective strategy for enabling high‐voltage ASSLMBs is the incorporation of fluorinated units, in the form of topologically engineered polymer matrices or bilayer membranes, which significantly improve oxidative stability against high‐voltage cathodes and enhance interfacial compatibility with lithium metal. However, the decreased ion conductivity and increased membrane thickness (exceeding 100 µm) offset this advantage.^[^ ^30^, ^31^, ^32^ ^]^ Moreover, fluorinated copolymers undergo complex processing steps and exhibit significant phase separation with PEO segments, thereby undermining their film‐forming capability.^[^ ^17^ ^]^ Furthermore, high‐concentration Li salts are widely employed for their capability to expand the electrochemical stability window of PEO‐based SSEs.^[^ ^33^ ^]^ The benefit is tempered by decreased ionic conductivity, increased costs, weakened mechanical properties, and a sharp increase in membrane viscosity and weight, collectively posing substantial challenges for large‐scale implementation. These approaches have yielded only modest performance improvement in low‐voltage LiFePO_4_ batteries.^[^ ^16^, ^34^ ^]^ Their poor cycling performance at high‐voltage ASSLMBs remains a significant barrier to practical application.

To effectively achieve advanced high‐voltage ASSLMBs, the following design principles should be considered: (i) Integration of nanoporous inorganic fillers into PEO‐based SSEs, leveraging the broad range of available nanoporous materials and their inherent interactions with the PEO matrix to enhance Li⁺ transport kinetics; and (ii) Selection of a diverse array of organic fluoroether segments, rather than inert perfluorinated alternatives, as weakened ion conducting groups. Building on these design principles, we hypothesize that engineering a weakly coordinated solvation structure can be established in PEO‐based SSEs, akin to the role of small‐molecule fluoroether solvents in ether‐based liquid electrolytes. As a conceptual design, a fluoroetherized titanium‐oxo cluster (FTOC) was employed as a demonstrative model, utilizing its microporous structure, coordination units analogous to the repeating structure of PEO matrices, and a rich array of labile surface ligands. After a simple solvent ligand exchange, the surface of the resulting FTOC can be progressively grafted with the fluoroether chains/groups. This strategy not only circumvents the reliance on high‐salt concentration methods, thick electrolyte architectures, and narrow operating temperature ranges, but also eliminates the necessity of incorporating the complex challenges of synthesizing single‐ion‐conducting polymers or inorganic superionic conductors into PEO‐based SSEs. As a result, we achieved an intrinsically antioxidative SSE, significantly enhancing oxidative stability up to 5.0 V. This design also improves other key properties, including high compatibility with lithium metal (stable Li plating/stripping over 9500 hours), ionic conductivity of 2.0 × 10^−4^ S cm⁻¹ at 30 °C, high toughness, reduced thickness of 20 µm, and high‐temperature full‐cell stability (100 °C). The synergistically enhanced properties of FTOC‐SSE enabled stable cycling in Li|FTOC‐SSE|LiNi_0.8_Co_0.1_Mn_0.1_O_2_ (NCM811) and Li|FTOC‐SSE|LiMn_0.6_Fe_0.4_PO_4_ (LMFP) cells. This work presents a low‐cost, efficient, and sustainable SSE design for high‐voltage ASSLMBs, paving the way for significant advancements in next‐generation high‐energy batteries.

## 2 Results and Discussions

### 2.1 Rational Design of PEO‐Based All‐Solid‐State Electrolyte for High‐Voltage ASSLMBs

**Figure 1:**
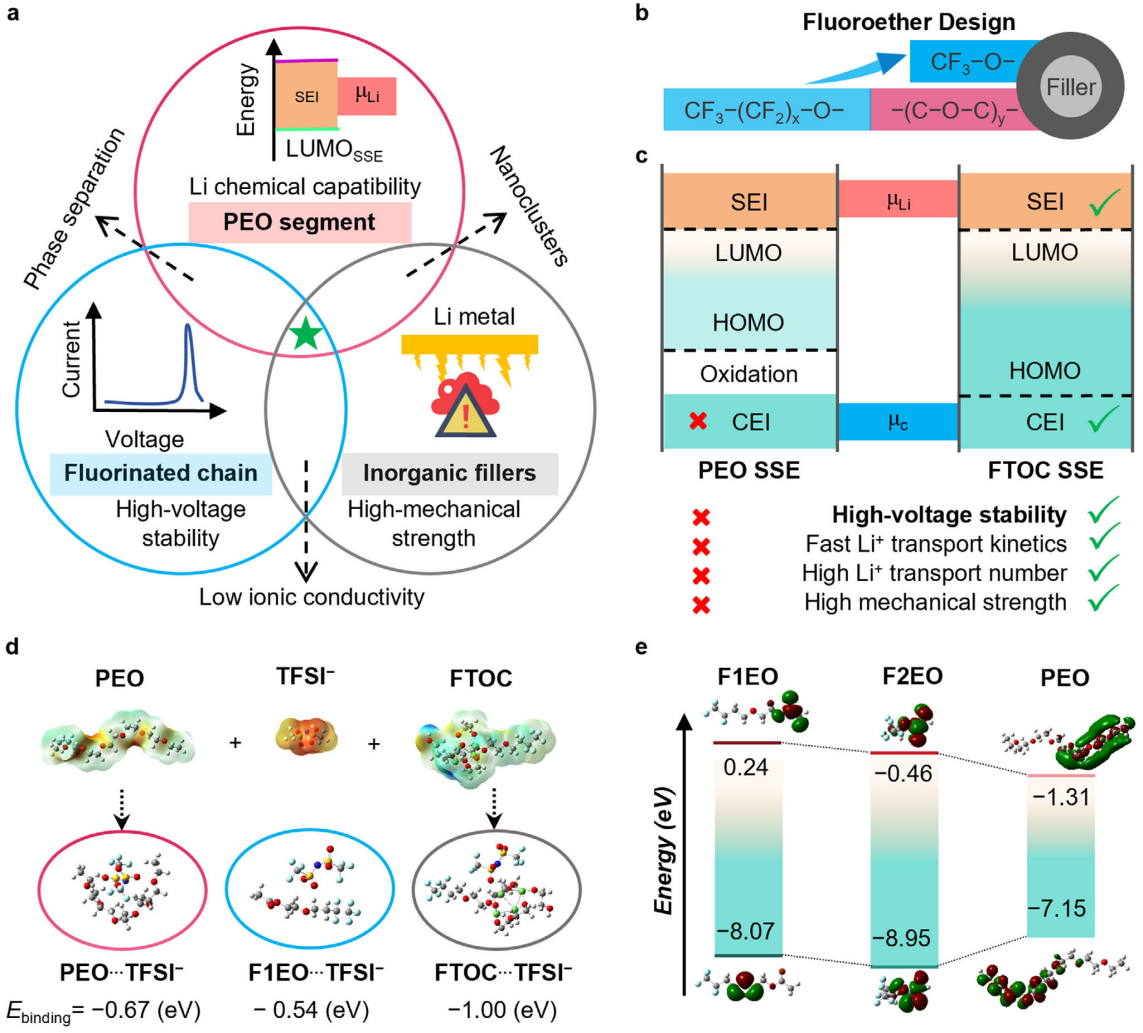
Schematic design principles for PEO‐based electrolytes in high‐voltage ASSLMBs. a) Design strategies of PEO‐based electrolytes for high‐voltage ASSLMBs. b) Fluoroether design for nanofillers with two typical kinds of CF_3_−O− short groups or CF_3_−(CF_2_)_x_−O−(C−O−C)_y_− long chains. c) Two different electrode/electrolyte interface models (left: a stable SEI passivating layer on the Li metal anode/SSE interface but unstable CEI on the high‐voltage cathode/SSE interface for PEO SSEs; right: a stable CEI passivating layer on the high‐voltage cathode/SSE interface and stable SEI on Li metal anode/SSE interface for FTOC‐SSEs). d) ESP results of PEO, TFSI^−^, and FTOC (red represents electronegativity, and blue represents positive charge); and calculated binding energy of PEO‐TFSI^−^, F1EO‐TFSI^−^, and FTOC‐TFSI^−^. Color code: grey, C; blue, N; red, O; yellow, S; and light green, Ti; pale blue, F. e) HOMO and LUMO energy levels of fluoroether chains of representative lengths (F1EO and F2EO represent long and short fluoroether segments, respectively) and PEO chain.

Superior antioxidation capability, enhanced ionic conductivity, and lithium metal compatibility are prerequisites for developing high‐voltage ASSLMBs. Figure 1a illustrates the electrolyte design principles that regulate Li chemical compatibility, ionic conductivity, and high‐voltage tolerance, in pursuit of high‐voltage solid‐state electrolytes (SSEs). Specifically, the PEO polymer exhibits exceptional lithium chemical compatibility, making it an ideal primary matrix for polymeric electrolytes. Unfortunately, simple interfacial modifications for PEO‐based SSEs provide only a short‐term extension of battery cycle life without compromising ionic conductivity, membrane thickness, and Li‐salt concentrations, suggesting that some trade‐offs must be made among key electrochemical performance parameters. As mentioned above, composite electrolyte engineering and the incorporation of fluoropolymers have emerged as two prevalent and effective strategies. Although incorporating fluoropolymers can broaden the electrochemical stability window, it inevitably results in severe phase separation and a decrease in ionic conductivity.^[^ ^30^ ^]^ Additionally, incompatible inorganic‐organic materials in composite electrolytes tend to form inorganic nanoclusters, which are limited by percolation effects, compromising electrolyte stability, especially at elevated temperatures.^[^ ^23^ ^]^ Furthermore, high‐voltage ASSLMBs experience significant “cross‐talking” effects within PEO‐based electrolytes, forming unstable solid‐electrolyte interphase (SEI) and cathode‐electrolyte interphase (CEI) layers.^[^ ^35^ ^]^

Based on our electrolyte design principle, we introduced a new type of organic‐inorganic nanofiller materials, as exemplified by fluoroetherized fillers, to efficiently integrate these three‐component materials into a unified system (marked as a green star in Figure 1a). As shown in Figure 1b, fluoroether chains/groups of varying lengths were grafted onto the surface of nanofillers. The nanoporous titanium‐oxo cluster (TOC) is an ideal candidate, owing to its surface ligands structurally analogous to those of the −C−O−C− units in PEO matrices and enriched with labile ligands. Their unique structure facilitates grafting through a simple and efficient stepwise ligand exchange strategy. Further details are described in Figure S1 (Supporting Information). Given their C−O−C‐containing segments, both fluorinated ether chains and TOC exhibit good miscibility with PEO, effectively suppressing phase separation. Such a hybrid nanofiller is expected to facilitate high‐voltage stability in PEO‐based SSEs while endowing PEO‐based SSEs with a comprehensive array of superior electrochemical properties beyond traditional PEO‐based SSEs, including fast Li^+^ transport kinetics and high mechanical strength (Figure 1c).

The high‐voltage stability of the fluoroether segments was validated by Density Functional Theory (DFT) calculations.^[^ ^17^ ^]^ As shown in Figure 1e, compared to pure PEO‐based SSEs (represented by polyethylene glycol, PEG),^[^ ^9^ ^]^ two typical types of esterified fluoroether alcohols (long‐chain F1EO and short‐chain F2EO) exhibit a significant decrease in the highest occupied molecular orbital (HOMO) energy due to chain fluorination. F2EO has the lowest HOMO energy level (−8.95 eV), indicating a high antioxidation ability that conforms to the design objectives shown in Figure 1c.^[^ ^5^, ^36^ ^]^ The proximity of F2EO to TOC enhances its electronic impedance, whereas the extended chain of F1EO improves its compatibility with PEO. Thus, the FTOC fillers contribute to optimizing both the electrochemical and phase stability of the composite. The DFT calculation results also reveal that the lowest unoccupied molecular orbital (LUMO) energies of F1EO and F2EO are higher than PEO polymer. The lower LUMO energy value indicates a greater propensity to accept electrons, facilitating electrochemical reduction. This indicates that both the long‐chain F1EO and the short‐chain F2EO segments have remarkable compatibility for interfacing with lithium metal anodes.^[^ ^37^ ^]^

From the initial conceptual design, we anticipated that the ordered porous structure of the nanofillers would facilitate Li⁺ transport by providing continuous and accessible ion‐conduction pathways. Therefore, further validation was required to assess the specific influence of this organic‐inorganic self‐assembly on Li^+^ transport efficiency. A comparative analysis of the electrostatic potential (ESP) and the binding energies of TFSI^−^ anions across the PEO polymer, F1EO, and F2EO was conducted by DFT calculations (Figure 1d; Figure S2, Supporting Information). The ESP results reveal a greater disparity in charge distribution between TFSI^−^ and FTOC compared to the PEO polymer, indicating that FTOC possesses a stronger ability to adsorb anions.^[^ ^38^ ^]^ In bond energy calculations, TFSI^−^ anions are affected by multiple interaction sites in the PEO segments, leading to a binding energy of −0.67 eV between PEO and TFSI^−^. For the F1EO‐TFSI^−^ system, fluorination reduces the bond energy between fluoroether molecules and TFSI^−^ anions, demonstrating a dilution effect analogous to liquid fluoroether solvents.^[^ ^39^ ^]^ In the FTOC–TFSI⁻ system, the binding energy with TFSI⁻ is the highest, indicating the strong Lewis acidity inherent to the TOC core, which serves to immobilize TFSI⁻ anions. In addition, FTOC exhibits two contrasting binding interactions with Li⁺, reflecting the dual effects of its molecular structure: the weak coordination nature of the fluoroether segments and the strong salt dissociation capability of the TOC core (Figure S2b. Supporting Information). The ability to dissolve LiTFSI salts has been further validated experimentally (Figure S3, Supporting Information). In addition, the LiTFSI dissociation energy decreases from 5.105 eV to 2.875 eV after FTOC incorporation. This differs from fluoroether solvents in liquid electrolytes, which primarily serve as diluents. Thus, from a theoretical perspective, our design principles are deemed feasible.

**Figure 2:**
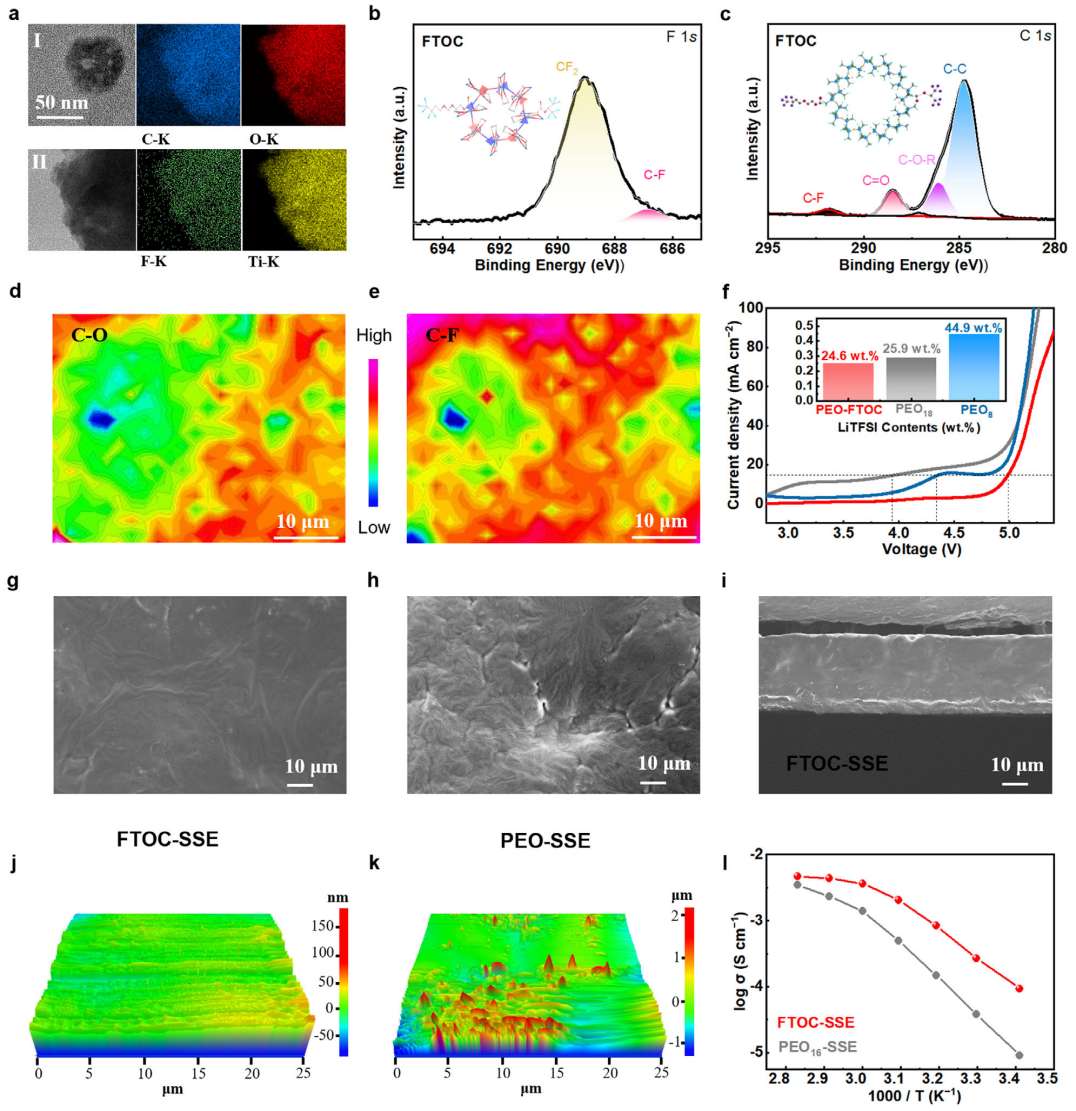
The effect of the fluoroether moiety presence in the composite polymeric electrolyte. a–c) Characterization of the nanoporous structures and the fluoroether functionalization, (a) TEM and elements mapping images of FTOC, containing C (blue), O (red), F (green), and Ti (yellow); and (b, c) The chemical structure of FTOC and XPS analysis of the characteristic peaks of FTOC: F 1s and C 1s spectra, respectively. d, e) Synchrotron‐based infrared mapping results of FTOC‐SSE in the range of (1122.80–1072.41 cm⁻¹ for C−O) and the TFSI⁻ anion (1218.50‐1166.50 cm⁻¹ for C−F). f) LSV results (the inset illustration shows Li‐salt concentration). g, h) Top‐surface SEM images of FTOC‐SSE and PEO‐SSE, respectively. i) Cross‐sectional SEM of FTOC‐SSE. j, k) 3D surface morphology of FTOC‐SSE and PEO‐SSE from AFM characterization. l) Ionic conductivities of FTOC‐SSE and PEO_16_‐SSE, showing the ionic conductivities in the temperature range from 20 °C to 80 °C.

The nanostructure of FTOC was characterized by transmission electron microscopy (TEM) and further confirmed by observation of the Tyndall effect (Figure 2a; Figure S4, Supporting Information). After the surface ligand exchange, the size of the FTOC nanofillers rapidly decreased to several tens of nanometers, with their surfaces exhibiting significant defects with a porous structure. The retention of the porous structure was further confirmed through N_2_ adsorption/desorption isotherms (Figure S5, Supporting Information). Furthermore, elemental mapping images show that the distribution of C and F extends beyond the nanoparticle boundaries, unlike the O and Ti elements, confirming the incorporation of F‐containing chain/group segments. The chemical structure of FTOC is shown in Figure 2b,c inner. Figures 2b,c, and S6 (Supporting Information) further demonstrate the successful grafting of characteristic fluoroether segments and the preserved chemical structure of the titanium‐oxo clusters, evidenced by X‐ray photoelectron spectroscopy (XPS) and Fourier transform infrared spectroscopy (FT‐IR). FT‐IR spectroscopy also reveals a significant reduction in the vibrational intensity of the −OH characteristic peak in FTOC, indicating the successful large‐scale exchange of surface‐bound deprotonated alcohol ligands. This also contributes to enhanced high‐voltage stability (Figure S7, Supporting Information).^[^ ^29^, ^40^ ^]^

The wide electrochemical stability window of SSEs is essential for pursuing the high‐energy‐density performance of ASSLMBs with high‐voltage cathodes. To validate the high‐voltage resistance of this design, linear sweep voltammetry (LSV) tests were conducted using Li||stainless steel (SS) cells. The oxidation potential of pure PEO‐SSEs has been verified to be ∼3.8 V vs. Li^+^/Li (Figure 2f). While high‐salt concentrations (EO: Li^+^ = 8:1) can improve the electrochemical stability of pure PEO‐based electrolytes, their leakage current density is still significantly unacceptable. By contrast, the oxidation potential of FTOC‐SSEs increases to ≈5.0 V. Additional electrochemical drift experiments and leakage current tests provide further evidence of the superior high‐voltage tolerance of FTOC‐SSEs (namely, no or negligible leakage current at high anodic polarization, see Figure S8, Supporting Information). FTOC‐SSE systems exhibit sustained stability at 4.6 V, whereas the PEO‐SSE undergoes significant decomposition at 4.0 V, resulting in a pronounced leakage current. Altogether, the FTOC‐SSEs with a wide electrochemical stability window exhibit substantial potential for compatibility with high‐voltage cathodes, which is crucial for enhancing the energy density of ASSLMBs. Due to the Li‐salt dissolution ability of FTOC and its excellent compatibility with the PEO‐based polymer matrix, the additive amount was optimized to be 25 wt% (relative to the polymer weight) without the need for additional Li salts. Therefore, the Li salt content is reduced to 24.6 wt.%, lower than that of conventional PEO‐based SSEs with an EO: Li⁺ ratio of 18:1 (25.9 wt.%) and significantly below the high‐salt condition of EO: Li⁺ = 8:1 (44.9 wt.%), as shown in Figure 2d (insert). Thus, the FTOC‐SSE exhibits excellent antioxidative stability even under low Li‐salt concentrations.

After the introduction of fluoroether segments and their substantial incorporation in PEO‐based SSEs, our initial hypothesis was that the high‐voltage stability of the synthesized FTOC‐SSEs primarily originated from changes in the Li^+^ coordination environment within the electrolyte. Synchrotron‐based infrared mapping was conducted to visualize the spatial distribution of LiTFSI and EO segments within the electrolyte. As shown in Figure 2d,e, mapping images corresponding to the characteristic C−O−C stretching vibrations (1050–1175 cm^−1^) and the TFSI⁻ anion (≈1210 cm^−1^) were obtained. The TFSI⁻‐rich regions are found to overlap with EO‐rich domains, and the broader distribution of TFSI⁻ signals further confirms that FTOC participates in the dissociation of LiTFSI. As a result, Li^+^‐TFSI^−^ exhibits a homogeneous distribution throughout the electrolyte. This homogeneous distribution contrasts with that in pure PEO‐based SSEs, where the coexistence of crystalline and amorphous phases often leads to spatially inconsistent Li⁺ dissociation. Raman spectroscopy was utilized to verify these alterations in the Li^+^ coordination environment within the electrolyte. According to the previous report, the peaks at approximately 738 and 742 cm^−1^ correspond to free TFSI^−^ anions, and the peak around 744 cm^−1^ is attributed to coordinated TFSI^−^ anions.^[^ ^29^, ^41^ ^]^ Figure S9 (Supporting Information) shows a pronounced increase in TFSI⁻ coordination peaks upon the introduction of FTOC. Moreover, an overall blue shift of these peaks indicates a greater incorporation of TFSI⁻ into the solvation sheath compared to PEO‐SSE matrices and PEO with other traditional oxide fillers.^[^ ^42^, ^43^ ^]^ Owing to the high FTOC content and the weak coordination ability of the fluorinated ether segments,^[^ ^17^ ^]^ the intensified ionic coordination observed in the Raman spectra is attributed to the dilution effect of FTOC on the PEO matrix. Furthermore, the ion aggregates are instead reduced. This is primarily due to the strong dissociation ability of the metal‐oxygen clusters toward LiTFSI, which is consistent with the synchrotron‐based infrared mapping results. The FT‐IR spectral results further support these findings, revealing distinct peak shifts and intensity changes associated with both LiTFSI and the PEO polymer backbone upon the introduction of FTOC. Furthermore, comparative FT‐IR analyses under varying lithium salt concentrations reveal a clear relaxation of C−O−C chain coordination in the FTOC‐SSE, indicating a reduced Li⁺−EO interaction resulting from both the fluorinated segment effects and the Lewis acidic nature of FTOC.^[^ ^44^ ^]^

To further understand the Li^+^ mobility in the FTOC‐SSE, ^7^Li solid‐state NMR spectra were measured (Figure S10a–c, Supporting Information). The dominant ^7^Li signal at ≈0.70 ppm in the FTOC‐SSE, which is shifted downfield compared to those in PEO‐SSE and PEO‐Oxide SSE, indicates a weaker Li⁺ coordination environment. Meanwhile, the sharp NMR peaks in the FTOC‐SSE system indicate rapid Li⁺ transport, whereas the broad peaks observed in PEO‐SSE suggest sluggish ion mobility. The results align with FT‐IR evidence of C−O−C chain relaxation, suggesting that FTOC suppresses C−O−C chain entanglement and facilitates Li⁺ mobility through a dynamically reorganized coordination environment. T_1_ measurements further confirm rapid ion transport at room temperature, compared to the PEO‐Oxide SSE (Figure S10d, Supporting Information). This confirms that fast ion transport arises not only from the filler‐induced acceleration effect. 2D‐Exchange Spectroscopy NMR offers selective and noninvasive insight into spontaneous Li⁺ migration across the composite interface. The clear presence of cross‐peaks at room temperature confirms efficient Li⁺ exchange across the two phases.^[^ ^28^, ^41^ ^]^ Thus, the incorporation of FTOC enhances Li^+^ and TFSI^−^ dissociation with weakened Li^+^ coordination chemistry.^[^ ^45^ ^]^ Conversely, in the case of the PEO‐Oxide SSE, the addition of oxide fillers results in a more constrained Li^+^ coordination environment than that observed in pure PEO‐SSE, attributed to the obstruction of the ion transport network arising from poor compatibility between the inorganic oxide particles and the polymer matrix.^[^ ^46^ ^]^

The influence of FTOC incorporation on other properties was thoroughly investigated to verify that the achieved high‐voltage stability did not compromise other critical electrochemical functionalities. The homogeneity of the FTOC‐SSE is observed in scanning electron microscopy (SEM) images (Figure 2g,i; Figure S11, Supporting Information), with further confirmation provided by EDS mapping. In contrast, SEM results of pure PEO membranes reveal spherical crystallites and porosity (see Figure 2i; Figure S11, Supporting Information). Figure S12 (Supporting Information) presents a surface SEM image of a PEO‐Oxide SSE, indicating that a high loading of oxide particles promotes nanoparticles clustering, resulting from phase separation. Atomic force microscopy (AFM) images confirm the smooth surface of the FTOC‐SSE membranes, with no visible signs of phase separation (Figure 2j). In contrast, pure PEO‐SSE samples exhibited a distinctly irregular morphology and straightforward evidence of phase separation at the micron scale (Figure 2k). Notably, unlike other approaches, the incorporation of FTOC significantly enhances the mechanical strength of the SSE, achieving a tensile deformation exceeding 250%, compared to only 150% for PEO‐SSE (Figure S13, Supporting Information). Furthermore, thermogravimetric analysis (TGA) demonstrates that adding FTOC results in a modest increase in decomposition temperature rather than a decrease (Figure S14, Supporting Information).

Although the incorporation of fluorinated polymers can significantly enhance the oxidative stability of the electrolytes, their inherent inertness toward Li^+^ conduction would lead to a decrease in the overall ionic conductivity. Fortunately, the introduction of FTOC is found to be beneficial for ion transport. The temperature‐dependent ionic conductivities of PEO‐SSEs and FTOC‐SSEs were measured and recorded as a function of temperature through the alternating current impedance method. As shown in Figure 2l and Figure S15 (Supporting Information), the addition of FTOC positively affects the ionic conductivity over the whole temperature range from 20 to 80 °C. The ionic conductivity of FTOC‐SSE matrices reaches 2.0 × 10^−4^ S cm^−1^ at 30 °C, exhibiting a significant improvement compared to the PEO‐based control samples (3.8 × 10^−5^ S cm^−1^). Furthermore, Figure S15 (Supporting Information) also provides comparison of ionic conductivities at different Li‐salt concentrations. Although high salt concentrations typically suppress ionic conductivity due to strong −C−O−C− coordination, the FTOC‐SSE maintains high conductivity even at an EO/Li⁺ ratio of 8:1. This confirms the decoupling effect of FTOC on PEO‐Li⁺ interactions, mitigating the chelation typically observed in molten PEO.

The mechanism underlying the enhanced ionic conductivity was investigated. Differential scanning calorimetry (DSC) was utilized to verify that the improvement in ionic conductivity arises from the decrease in crystalline regions (Figure S16 and Table S1, Supporting Information), as reflected by the shifts observed in the melting temperature (T_m_). FTOC delivers the lowest T_m_, indicating an increased proportion of its amorphous regions. The crystallinity calculated from the exothermic enthalpy further confirms that the introduction of FTOC significantly reduces the degree of crystallinity (Table S1, Supporting Information). X‐ray diffraction (XRD) further confirms that amorphous regions are predominant in FTOC‐SSE matrices (Figure S17, Supporting Information). The XRD characteristic peaks associated with the crystalline phase of the PEO matrices at 2*θ* = 19.2° and 23.3° are absent in the patterns of FTOC‐SSE matrices, suggesting a predominance of the amorphous phase.^[^ ^47^ ^]^

**Figure 3:**
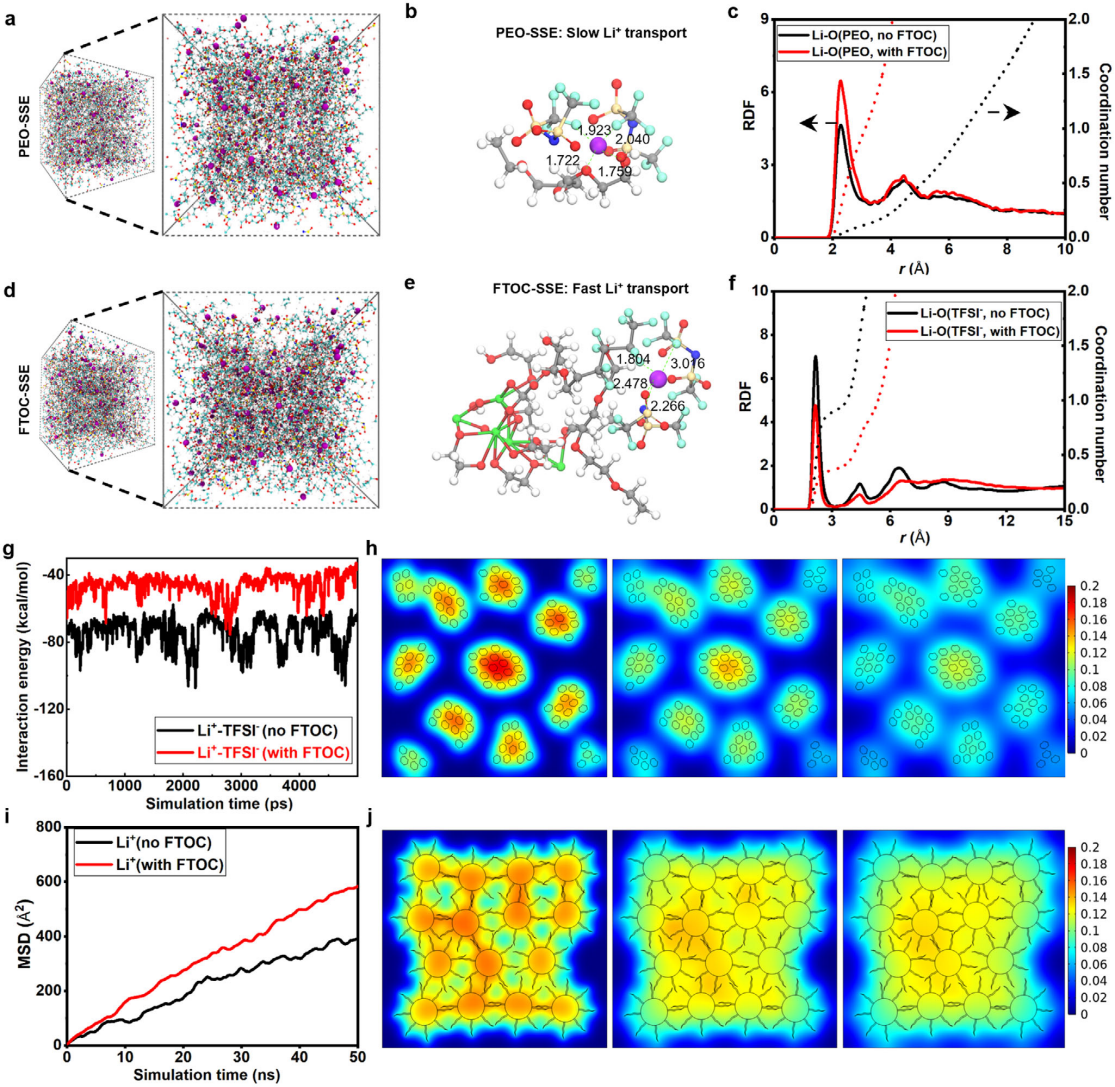
Various theoretical calculations and characterizations of Li^+^ coordination environment changes and their impact on ion transport in PEO‐based SSEs with and without FTOC. Representative snapshots of molecular dynamic (MD) simulation: a) for PEO‐SSE, and d) for FTOC‐SSE (grey, Ti; cyan, C; red, O; light green, F; and purple, Li). Snapshots obtained from MD simulations of b) PEO‐SSE and e) FTOC‐SSE. DFT calculations: c) Calculated RDF and coordination number of Li^+^‐O (PEO), and f) Li‐O (TFSI^−^). g) The interaction energy of Li^+^‐TFSI^−^; and i) Calculated MSD of Li^+^. The formation mechanism of uniform Li^+^ ions flux. FEMS results of Li^+^ transport mode in the h) PEO‐oxide SSE and j) FTOC‐SSE. The right scales show Li^+^ ions concentration (mol L^−1^).

To gain deeper insight into the origins of the high‐voltage stability exhibited by the FTOC‐SSE matrices, we conducted molecular dynamic (MD) simulations to describe the altered coordination environment with/without FTOC in PEO‐based SSEs as a proof‐of‐concept.^[^ ^48^, ^49^ ^]^ The specific parameters of the MD simulations are detailed in the Supporting Information. Figure 3a,d are representative snapshots of the simulation process, which corresponds to pure PEO‐SSE and FTOC‐SSE, respectively. Introducing FTOC increases the Li^+^ concentration within the PEO‐rich electrolyte system (Figure 3d). Radial distribution function (RDF) analysis and coordination number calculations were conducted to provide a detailed understanding of the intrinsic localized coordination structures at a molecular level. The results reveal that in the pure PEO‐SSE, Li^+^ normally interacts with multiple −C−O−C− bonds and TFSI^−^ (S═O bonds), as shown in Figure 3c,f. Li‐O coordination peaks for PEO in FTOC‐SSE are elevated, compared with Li‐O coordination peaks for PEO in PEO‐SSE, across all coordination layers. Additionally, the coordination number of Li‐PEO has significantly increased in the FTOC‐SSE.^[^ ^50^ ^]^ Additionally, the calculated coordination numbers for Li−O and Li−N (TFSI⁻) exhibit a decreasing trend, accompanied by a noticeable weakening of Li^+^‐TFSI^−^ interactions, further confirming the effective dissociation of the lithium salt (see Figure 3f,g; Figure S18, Supporting Information).^[^ ^44^ ^]^

Further investigation indicates that the slope of the curve of Li^+^ ions diffusion in FTOC‐SSE is significantly higher than that in PEO‐SSE in the calculation of the mean‐squared displacement (MSD, Figure 3i), attributed to FTOC having more rapid Li^+^ ions diffusion kinetics due to the decoupling of Li^+^‐PEO coordination induced by the dilution effect. As illustrated in Figure 3b,e, the representative configuration reveals that Li⁺ coordinates with both C−O−C backbone oxygen atoms and TFSI⁻ anions. In the FTOC‐SSE system, the Li⁺‐PEO distance increases from 1.722/1.759 Å to 2.266 Å, indicating a weakened Li⁺‐PEO interaction. Meanwhile, the Li⁺−O distance in TFSI⁻ also increases significantly, from 1.759 Å to 3.016 Å. Thus, a reorganized Li^+^ solvation structure is established. In the FTOC system, fluorinated ether chains participate in Li⁺ solvation. But, due to their perfluorinated end groups, the incorporation of FTOC increases the Li⁺ coordination number toward PEO segments with a high donor number, as supported by Raman and RDF results. Meanwhile, FTOC's strong anion adsorption, reduced salt dissociation energy, and increased Li⁺ coordination distances in Li^+^‐EO confirm a weakly coordinated environment, consistent with SS‐NMR observations.

Finite element method simulations (FEMS) using *COMSOL Multiphysics* were conducted to elucidate the Li⁺ ions transport mechanism within the PEO‐oxide SSEs and FTOC‐SSEs. The simulations examine Li⁺ ion flux around the filler particles.^[^ ^51^ ^]^ Strong Lewis acid‐base interactions between the oxide particles and Li⁺ ions enhance Li⁺ adsorption, resulting in high Li⁺ ion concentration regions around the oxide nanoclusters in the initial state (Figure 3h). Under an electric field, Li⁺ ions leave these oxide nanoclusters and migrate within the PEO electrolyte. Unfortunately, due to the promotion of Li‐salt dissociation by oxide particles, the Li⁺ ion concentration within the oxide nanoparticles remains higher than that in the surrounding PEO electrolyte, causing an uneven distribution of free Li⁺ ions. In contrast, uniform Li⁺ ions transport is observed along the interface and partially within the FTOC interior, forming numerous long‐range continuous Li⁺ ion transport channels (Figure 3j). The presence of fluoroether “brushes” creates continuous pathways that can facilitate both Li⁺ ion reception and delivery across FTOC nanofillers within the PEO‐based electrolyte. Figure S19 (Supporting Information) also compares the ionic diffusion coefficients in the FTOC‐SSE and PEO‐Oxide SSE systems. In the FTOC‐SSE, the FTOC region exhibits an ionic diffusion coefficient approximately 15 times greater than that of the PEO matrix, and notably higher than the oxide phase in the control sample, which shows only a 5‐fold increase relative to the PEO phase. This observation suggests that the FTOC domains facilitate fast ion transport, possibly due to the formation of a less coordinated and more dynamic Li⁺ environment. Consequently, the Li⁺ ion concentration exhibits a significantly uniform distribution in the FTOC‐SSE.

### 2.2 Compatibility of Li Metal Anodes

**Figure 4:**
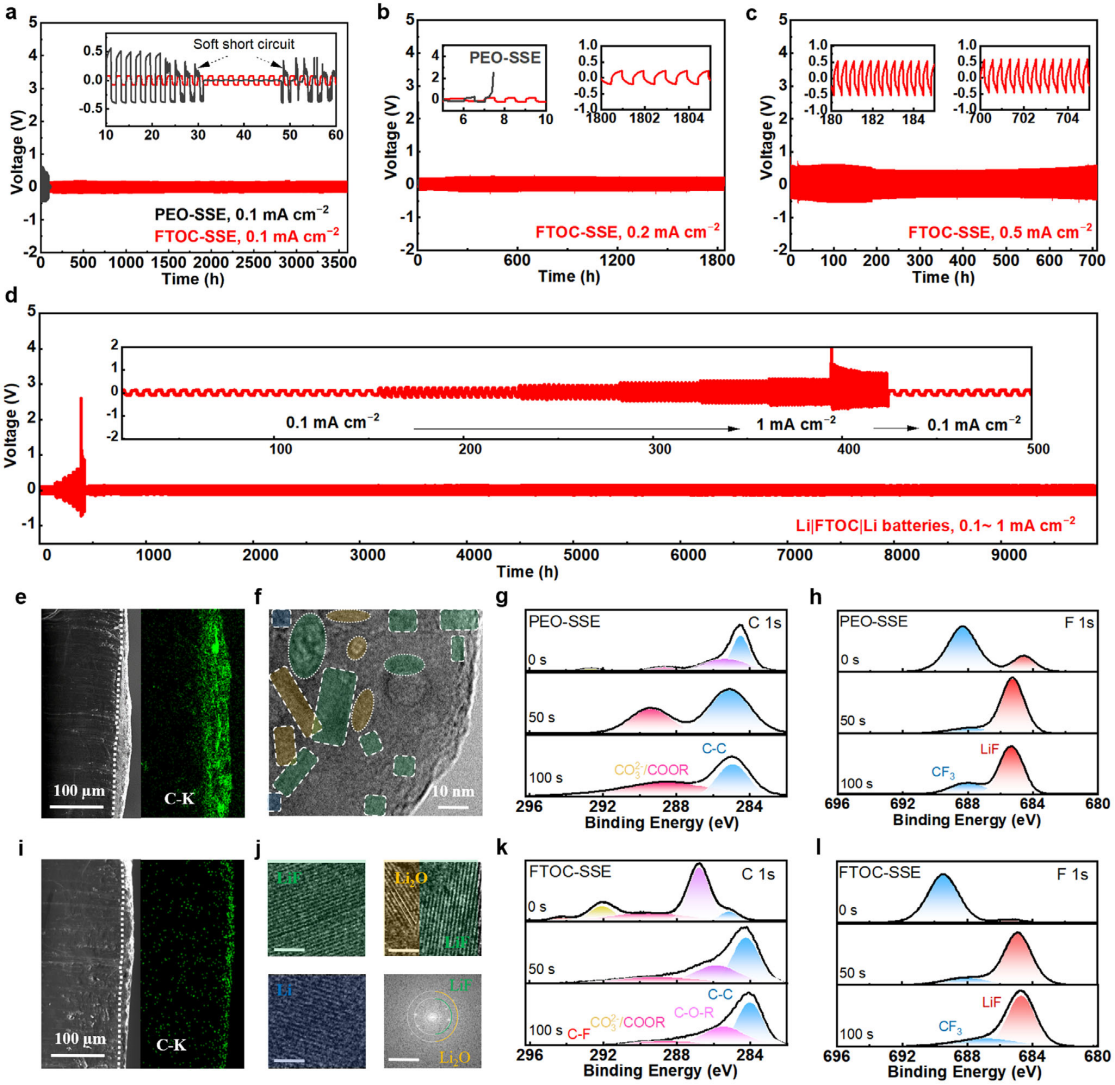
Electrochemical performance of Li||Li symmetric cells and the resultant lithium metal deposition morphology. Galvanostatic cycling performance at different current densities a) Li||Li at 0.1 mA cm^−2^, b) 0.2 mA cm^−2^, and c) 0.5 mA cm^−2^. d) Various current densities and galvanostatic cycling performance of Li||Li cells at 0.1–1 mA cm^−2^ at 60 °C. SEM‐EDS mapping analysis after 100h e) for Li electrodes from cells based on pure PEO‐SSE and i) for Li electrodes from cells based on FTOC‐SSE. f) Cryo‐TEM characterization of Li electrodes taken from Li|FTOC‐SSE|Cu cells after 50 h, j) magnified images of nanocrystals in (f) and corresponding FFT image. Lattice fringes of Li_2_O and LiF can be observed in (j). XPS results related to C 1*s* g) measured for Li anodes taken from cells based on pure PEO‐SSE and k) FTOC‐SSE; and spectra related to F 1*s* measured with Li anodes taken from cells based on h) pure PEO‐SSE and on l) FTOC‐SSE.

The electrochemical compatibility of FTOC‐SSE with lithium metal anodes was initially examined through the elucidation of Tafel plots, which confirms a significantly increased exchange current density in Li|FTOC‐SSE|Li cells compared to Li|PEO‐SSE|Li cells at room temperature (Figure S20, Supporting Information). It suggests that FTOC‐SSE can increase the interfacial rate of charge transfer.^[^ ^9^ ^]^ Rate capability and long‐cycle testing of the symmetric lithium batteries were conducted to rapidly screen optimized components. Further details are provided in Figure S21 (Supporting Information). Figures 4a and S22 (Supporting Information) demonstrate that the stable Li plating and stripping processes could last at least 3500 h at 0.1 mA cm^−2^ and 0.1 mAh cm^−2^ with a unilateral overpotential as small as 90 mV. By contrast, Li cells based on pure PEO‐SSE undergo rapid soft short‐circuiting, attributed to their low ionic conductivity and an inhomogeneous structure of the SSE resulting from pronounced phase separation. Under 0.2 mA cm^−2^, cells based on PEO‐SSE fail to operate due to excessive polarization. In contrast, Li cells based on FTOC‐SSE demonstrate stable operation at a current density of 0.2 and 0.5 mAh cm^−2^, as shown in Figure 4b,c. At an elevated operating temperature of 60 °C, Li cells based on PEO‐SSE remain prone to short‐circuiting as this temperature exceeds their melting point (Figure S23, Supporting Information). Significant voltage polarization is observed with cells based on PEO‐oxide SSE when the current density was raised to 0.4 mA cm^−2^ (Figure S24, Supporting Information). For unmodified oxides, filler loading equivalent to that used in FTOC systems is considered excessively high and detrimental to electrolyte performance. They induce the rapid short‐circuiting of the cells, primarily due to excessive nanoparticle clustering. In contrast, Li cells based on FTOC‐SSE could be operated at high current density (1 mA cm^−2^), sustaining stable operation for over 9500 h even with electrolyte membranes as thin as 20 µm (Figure 4d).

SEM images of the cycled Li electrode surfaces were used to investigate the lithium deposition behavior at 60 °C. Prominent Li dendrites formation is observed with Li anodes taken from cells based on PEO‐SSE, consistent with the cycling performance of the relevant symmetric lithium metal batteries (Figure S25, Supporting Information). In cells with PEO‐SSE, Li deposition displays an irregular, porous morphology, largely attributed to the loss of mechanical strength of the polymeric electrolyte membranes at elevated temperatures, as shown in Figure 4e. For the case of Li cells based on FTOC‐SSEs, analysis of Li electrodes’ cross‐sections by SEM imaging reveals that, even at the elevated temperature condition, the Li deposition morphology retains a consistently dense and uniform compact structure (Figure 4i).

To gain deeper insight into the morphology and composition of the SEI‐type surface films formed on the Li anodes, multiscale cryogenic transmission electron microscopy (Cryo‐TEM) characterization was conducted. Analysis of cross‐section views of Li anodes reveals thick, amorphous, and organic‐rich layers for electrodes taken from cells based on PEO‐SSE (Figure S26, Supporting Information). Low‐magnification Cryo‐TEM reveals irregularities on the Li deposit's surface, while high‐magnification Cryo‐TEM confirms that the highly ordered crystalline Li (110) is in direct contact with the amorphous organic layer. In contrast, for Li anodes taken from cells based on FTOC‐SSE, the microscopic imaging exhibits uniform Li deposition layers with distinct texture striations (Figure S27, Supporting Information). This comparison suggests that the pure PEO‐based SSE facilitates the formation of surface layers on the Li electrodes composed of organic components. In turn, FTOC‐based SSE promotes the formation of thin inorganic‐enriched surface (SEI type) layers on the Li electrodes, effectively suppressing lithium dendrite formation.^[^ ^52^ ^]^ A detailed analysis of surface layers formed on Li electrodes in contact with FTOC‐SEE was obtained by high‐resolution Cryo‐TEM imaging (Figure 4f,j). The lithium metal surface is coated with abundant inorganic nanocrystals. The outer layer exhibits an amorphous structure with sparse nanocrystalline domains, while the inner layer is rich in nanocrystalline regions with clearly resolved lattice fringes. The presence of highly ordered crystalline particles is evident in Figure 4j. Nanocrystals were categorized by color based on their different lattice spacings. Given that the innermost region corresponds to metallic lithium (turquoise), the adjacent crystalline phase is assigned to Li_2_O (yellow), while the nanocrystals enriched in the outer region are attributed to LiF (green). The lattice spacings corresponding to LiF and Li_2_O are identified by the associated fast Fourier transform (FFT) image. Therefore, it can be concluded that incorporating FTOC into the PEO‐based SSEs effectively suppresses Li‐dendrite formation, leading to the formation of a stable SEI on the Li metal electrodes in cells based on FTOC‐SSE.

XPS measurements were performed after Cs^+^ sputtering for 0, 50, and 100 s on the surface films of the Li anodes. Compared to cells based on PEO‐SSE (Figure 4g,h), the X‐ray photoelectron spectra + sputtering measured with cycled Li anodes in cells based on FTOC‐SSE show slight changes in the C and F XPS peaks upon sputtering, indicating that the surface films are stable (Figure 4k, l).^[^ ^53^, ^54^ ^]^ Conversely, the O content rapidly decreases upon spattering from 23.19% (0s) to 7.34% (50s), suggesting that the outer surfaces in the surface films formed on the Li anodes are enriched with a thin organic layer when the cells are based on FTOC‐SSE (Figure S28, Supporting Information). This is beneficial in regulating Li^+^ ion diffusion and inducing the uniformity of Li deposition, which has often been attributed to LiF‐rich surface films that act as structurally stable SEI‐type passivation layers on the lithium metal electrodes. Figure S29 (Supporting Information) further reveals a shift in the XPS N 1s peak from the characteristic position of LiTFSI to that of Li_3_N (upon sputtering the measured Li surfaces), indicating that the introduction of FTOC as a central moiety in the SSE facilitates the favorable decomposition of LiTFSI.^[^ ^47^ ^]^ In contrast, in control (reference) experiments related to Li cells with pure PEO‐based SSE, the LiTFSI‐related N 1s peak disappears with extended etching time (by sputtering), suggesting that the relevant surface films formed on the Li electrodes taken from these cells are composed exclusively of organic species.

### 2.3 Electrochemical Performance of High‐Voltage ASSLMBs

**Figure 5:**
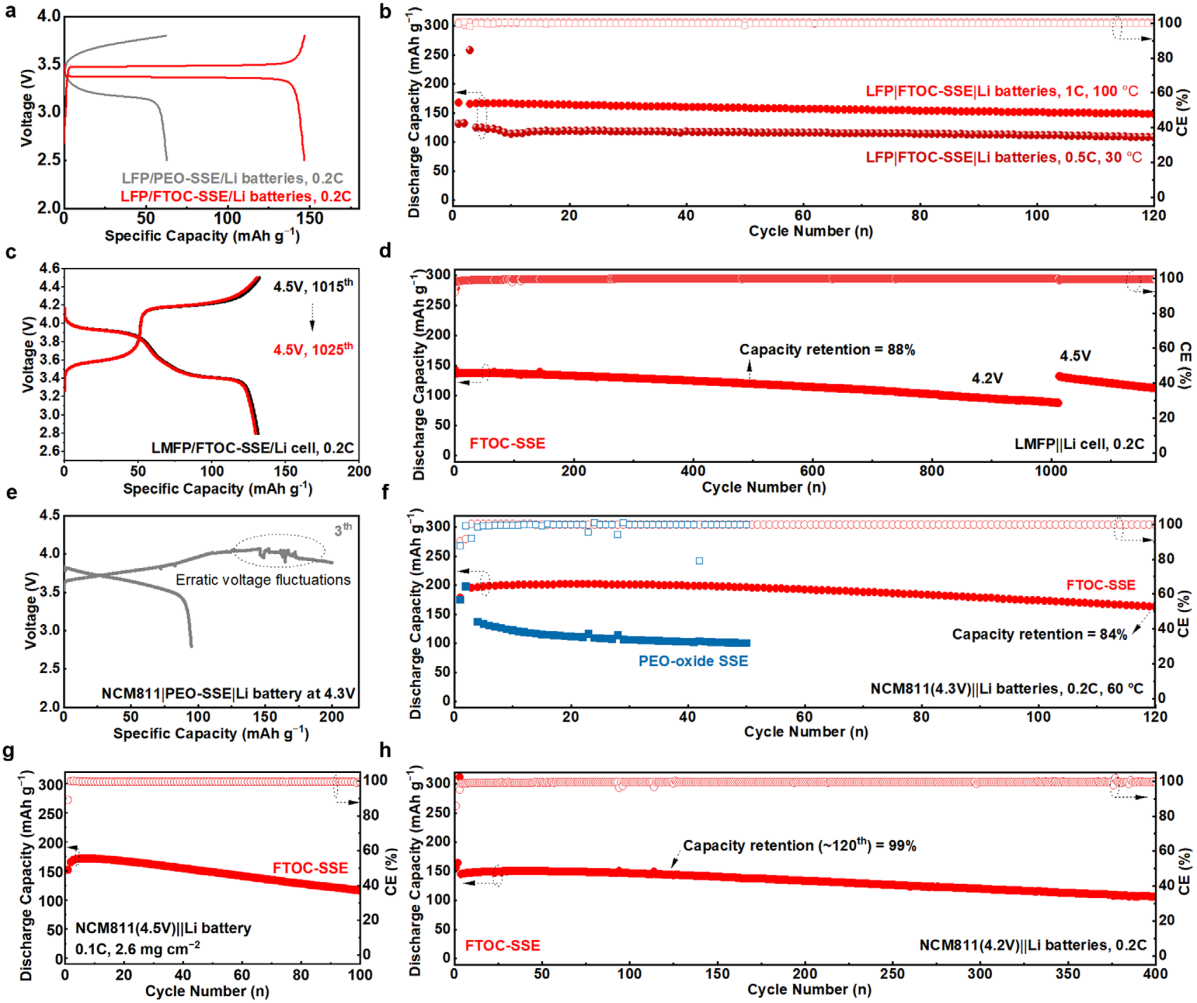
Electrochemical properties of ASSLMBs with FTOC‐SSE, tests based on galvanostatic experiments. a) Comparison of charging and discharging voltage profiles for Li|FTOC‐SSE|LFP and Li|PEO‐SSE|LFP batteries at 50 °C. b) Different tests’ temperatures for Li|FTOC‐SSE|LFP batteries (30–100 °C). c,d) Charging and discharging curves of Li|FTOC‐SSE|LMFP batteries during cycling with a cut‐off voltage of 4.5 V and their cycling stability at 0.2C. e) Charging and discharging curves of Li|PEO‐SSE|NCM811(4.3 V) cells. f) Comparison of cycling stability for Li||NCM811(4.3 V) batteries at 60 °C. (g,h) Long‐cycling performance of Li|FTOC‐SSE|NCM811 cells at 50 °C with a cut‐off voltage of 4.5 V and 4.2 V, respectively.

Before high‐voltage full‐cells testing, LiFePO_4_ (LFP)||Li full cells were selected to assess full‐cells stability. A comparison of polarization voltage during charge/discharge cycles is exhibited in Figure 5a, demonstrating that cells with PEO‐SSE deliver high polarization voltage because of the low conductivity of their solid electrolyte at 50 °C. By comparison, LFP|FTOC‐SSE|Li batteries demonstrate relatively low voltage polarization, with an initial capacity reaching 148.4 mAh g⁻¹, as well as notable long‐term cycling stability (Figure S30, Supporting Information). Figure 5b shows the stable cycling performance of LFP|FTOC‐SSE|Li cells over a wide temperature range (from 30 to 100 °C). In contrast, LFP|PEO‐SSE|Li cells show a typical electrolyte decomposition at 100 °C. Furthermore, the cells containing PEO‐oxide SSE exhibit a sharp increase in their overpotential at 100 °C, resulting from the formation of severe nanoclusters at that elevated temperature (Figure S31, Supporting Information).

For high‐voltage full‐cell examination, LMFP|FTOC‐SSE|Li cells with increasing cut‐off voltages were evaluated at 0.5C to verify the ultra‐durable antioxidant performance of the FTOC‐SSEs. As shown in Figure S32 (Supporting Information), these cells exhibit minimal capacity fading with discharge capacity retention of 99% after 500 cycles upon charging to 4.2V. As the voltage gradually increased from 4.2V to 4.5V, the cells’ capacity recovers to its initial level, demonstrating the durability of these battery prototypes under high voltage. Even without any cathode surface treatment or structural framework, both the LMFP full cells and Li||Li symmetric cells exhibit stable cycling performance using FTOC‐SSE, as shown in Figure S32 (Supporting Information). The result suggests that the enhanced electrochemical stability primarily originates from the introduction of FTOC.

Furthermore, the high‐voltage LMFP|FTOC‐SSE|Li cells cycled at 0.2C and charged up to 4.2 V also exhibited superior cycling stability, as shown in Figure 5d. After undergoing over 1000 cycles without any decomposition, the cell's cutoff voltage was raised to 4.5 V. Remarkably, their capacity recovered to over 96% of its initial value, while maintaining stable cycling performance. Figure 5c shows the LMFP cathode exhibiting two distinct voltage plateaus at ≈3.5 V and ≈4.2 V, corresponding to the Fe^2^⁺/Fe^3^⁺ and Mn^3^⁺/Mn^4^⁺ redox couples, respectively. The clear plateau features indicate effective utilization of both redox reactions and good interfacial compatibility in the FTOC‐SSE system.

To further confirm the exceptionally high‐voltage stability of FTOC‐SSE, single‐crystal LiNi_0.8_Co_0.1_Mn_0.1_O_2_ (NMC811)||Li full cells were assembled. The cells were first assessed at 60 °C (above T_m_) to mitigate bad influences caused by low operating temperatures. Figures 5e and S33 (Supporting Information) display that PEO‐SSE‐based cells with the same electrodes encounter sporadic decomposition when NMC811 (4.3 V)||Li full cells were tested, consistent with previous studies.^[^ ^11^, ^12^ ^]^ The PEO‐oxide SSE exhibits increased voltage polarization and electrochemical instability from the fifth cycle, resulting in significant decomposition during less than 30 cycles with NCM811|PEO‐SSE|Li cells (Figure S34, Supporting Information). In contrast, NMC811(4.3 V)|FTOC‐SSE|Li cells exhibit an initial capacity of 190 mAh g⁻¹ at 0.2C with a first‐cycle Coulombic efficiency of 92%, maintaining electrolyte stability without decomposition over 100 cycles (Figure 5f). Stable long‐term cycling performance was further achieved when the operating temperature of the full cells was reduced to 50 °C or 30 °C (Figure 5h; Figure S35, Supporting Information). NMC811|FTOC‐SSE|Li cells retain 99% discharge capacity at 0.2C after 120 cycles. Figures 5g and S36 (Supporting Information) illustrates that the NCM811 battery operates reliably at 4.5 V, confirming its exceptional antioxidative stability with high‐nickel cathode materials.

**Figure 6:**
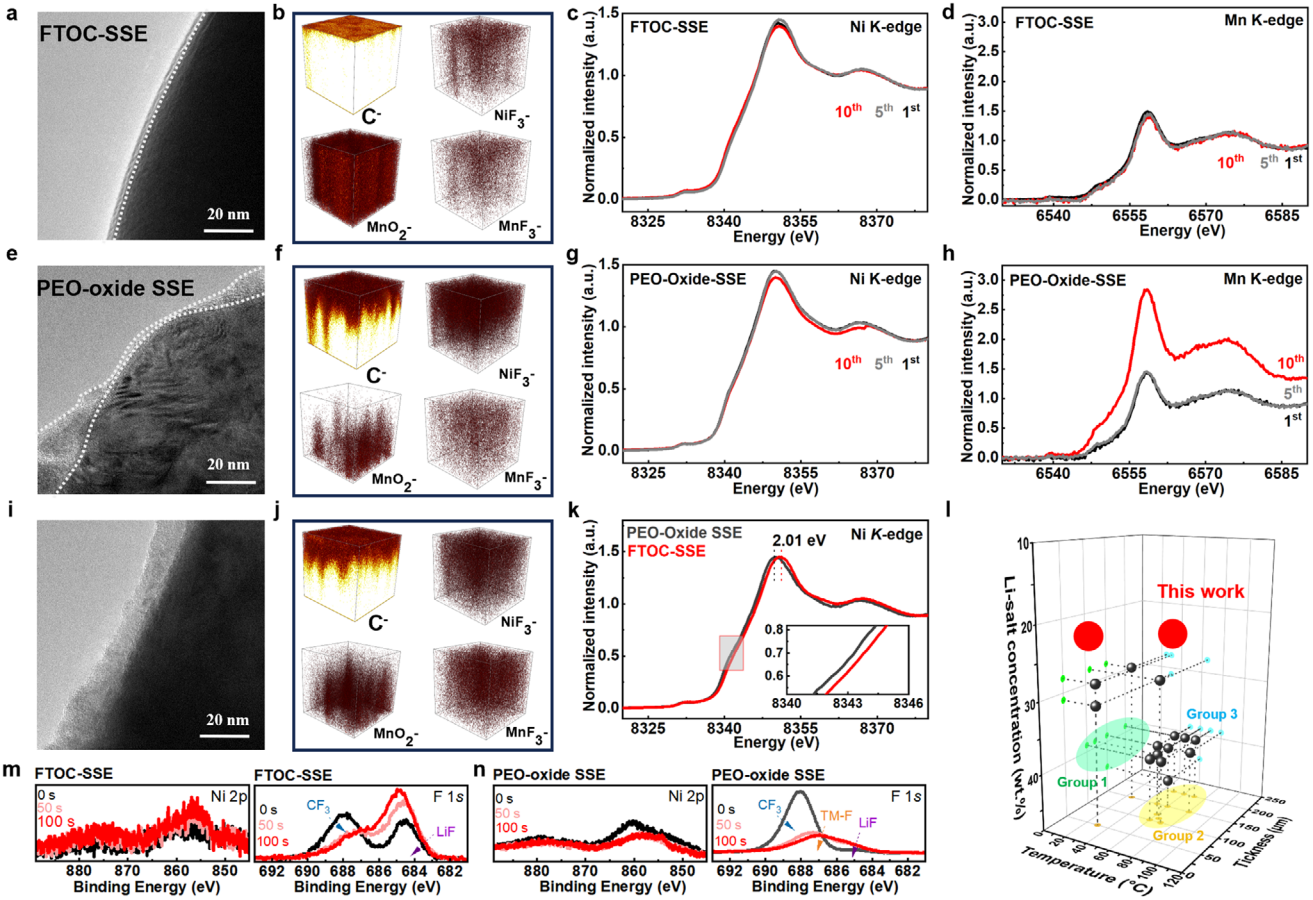
Various characterizations of cathodes and related CEI layers after being cycled in cells based on various types of SSE. a,e, and i) TEM images of NCM811 cathodes from cells based on FTOC‐SSE (10 cycles), PEO‐oxide SSE (10 cycles), and PEO‐oxide SSE (50 cycles), respectively. b,f, and j) 3D ToF‐SIMS results of NCM811 cathodes from cells based on FTOC‐SSE (10 cycles), PEO‐oxide SSE (10 cycles), and PEO‐oxide SSE (50 cycles), respectively. c,g, and k) XANES Ni *K*‐edge spectra measured with cathodes upon cycling in cells based on FTOC‐SSE, PEO‐oxide SSE, and FTOC‐SSE/PEO‐oxide SSE (locally enlarged image, 10^th^ cycle), respectively. The cycle numbers are indicated therein. d,h) XANES Mn *K*‐edge spectra measured with cathodes upon cycling in cells based on FTOC‐SSE, and PEO‐oxide SSE, respectively. l) Comparison with previous works regarding low lithium salt concentration, wide operating temperature range, and thin film formation. m) In‐depth XPS Ni 2p and F 1s peaks obtained by measuring cathodes after being cycled in cells based on FTOC‐SSE, and n) and on PEO‐oxide SSE.

To study the mechanism of FTOC‐SSE stabilization of high‐voltage cathode materials, TEM measurements were performed to investigate the morphology of the surface films formed on NCM811 cathodes (also denoted as CEI, cathode electrolyte interphase) after cycling. Cells based on pure PEO‐SSEs and PEO‐oxide SSEs, which demonstrate rapid failure, were used as reference systems for further comparison. After ten cycles, a thin and uniform CEI of ≈3 nm is formed on NCM811 particles when cycled in cells with FTOC‐SSE (Figure 6a). In contrast, thick and heterogeneous CEI layers (0–20 nm) are formed on NCM811 particles when the cathodes are cycled 10 times in cells containing PEO‐oxide SSE. (Figure 6e). A comparative analysis of CEI formation reveals that after 50 cycles, the cathodes in cells based on PEO‐oxide SSE develop a CEI with thicknesses over 20 nm (Figure 6i). In turn, similar cathodes, cycled 50 cycles in cells based on FTOC‐SSE, form a more uniform CEI layer with a thickness of less than 10 nm (Figure S37, Supporting Information). Time‐of‐flight secondary ion mass spectrometry (ToF‐SIMS) was used to visualize the CEI constructions and sedimentation. Cycled cathodes retrieved from NCM811||Li cells after 10 cycles were studied (Figure 6b,f; Figure S38, Supporting Information). The decomposition concentration gradient is highly pronounced and uneven when the cells contain PEO‐oxide SSE. In contrast, when the cells contained FTOC‐SSE, thin and homogeneous surface layers are formed. Additionally, the concentration of transition metal fluorides (TM‐F) found in the cathodes’ surface films after being cycled with cells based on FTOC‐SSE are markedly lower than their content in the surface films formed on cathodes cycled in the cells based on PEO‐oxide SSE.^[^ ^55^, ^56^ ^]^ To elucidate the decomposition mechanism in PEO‐oxide SSE‐based cells, cathodes after 50 cycles were further investigated. The results indicate that, as the cycle of cells with MCM 811 cathodes and PEO‐oxide SSE progresses, Ni‐F moieties are preferentially formed, subsequently followed by an accelerated formation of Mn‐F compounds (Figure 6i).

Although higher Ni content improves the specific capacity of cathode materials, it is accompanied by the irreversible reduction of Ni^4+^ ions in their highly lithiated state to Ni^2+^ ions through side reactions with solution species, which are oxidized by the Ni ions at their high oxidation state.^[^ ^57^ ^]^ These Ni^2+^ ions subsequently migrate into the Li layers with the cathodes’ particles, leading to Li/Ni cation mixing and a phase transition from the layer structure to a rock‐salt phase of these NCM cathode materials. Highly reactive transition metal (TM) ions readily interact with SSE, leading to the formation of irreversible TM‐F products. The subsequent dissolution of TM ions further accelerates this reaction pathway. X‐ray absorption near‐edge structure (XANES) using high‐energy (“hard”) X‐rays can effectively penetrate these Ni‐rich cathode materials, making it a powerful tool for investigating the overall TM‐ion valence states and their coordination environment. As indicated in Figure 6c,g, the Ni *K*‐edge spectra of cathodes cycled 10 times in cells based on both PEO‐oxide SSE and FTOC‐SSE measured while being charged to 4.3 V, maintained unchanged energy peak positions from the 1^st^ to the 10^th^ cycle without a significant indication for structural changes. However, as presented in Figure 6i, the enlarged Ni‐*K* edge spectra of fully charged NCM811 cathodes in cells based on PEO‐oxide SSE were shifted to a lower energy position compared with the cathodes cycled in cells based on FTOC‐SSE, suggesting that the overall valence of Ni ions was decreased in the latter case, which could be explained by spontaneous Ni^3+/4+^ reduction by interaction with the PEO‐oxide SSE. It demonstrates that the high oxidation state of Ni in charged NCM811 cathodes in cells containing FTOC‐SSE is much more stable than in cells containing PEO‐oxide SSE. Hence, the utilization and reversibility of the Ni^3+^/Ni^4+^ redox reactions in Ni‐rich NCM cathodes are significantly improved in the former case, owing to the robust CEI structure when FTOC‐SSE is used in solid‐state battery prototypes. Compared with the similar spectra related to the NCM cathodes cycled in the cells based on PEO‐oxide SSE from the 1^st^ to the 10^th^ cycle, the almost unchanged Ni *K*‐edge spectra of the NCM cathodes cycled in the cells based on the FTOC‐SSE demonstrates that the contact with the FTOC‐SSE improves the reversible flexible distortion of the NiO_6_ octahedra in the layered‐structure NCM cathodes and mitigates anti‐site cations mixing. (Figure S39a,b, Supporting Information).^[^ ^58^ ^]^ XAS characterization of the cycled electrodes reveals a 2.01 eV shift toward lower energy, indicating significant structural collapse and substantial Ni reduction after cycling in traditional PEO‐oxide‐based electrolytes (Figure 6k). These spectral results reflect an average Ni valence in these layered NCM cathodes, which depends on the nature of the solid electrolyte used. The Ni valence in the cathodes cycled in the cells based on PEO‐oxide SSE is lower than the Ni valence of the NCM cathodes cycled in cells based on the FTOC‐SSE. Furthermore, Mn *K*‐edge spectra of both types of NCM811 electrodes (defined by the different SSE used) measured at different cycles are given in Figure 6d,h. The spectra of the NCM cathodes cycled in the cells based on PEO‐oxide SSE change significantly at the 10^th^ cycle, which also indicates that the Mn ions have migrated to the Li^+^ ions layers.^[^ ^59^ ^]^ The stable Mn *K*‐edge spectra of the NCM cathodes cycled in the cells based on FTOC‐SSE indicate well that the use of FTOC which increases pronouncedly the anodic stability of the electrolyte system preserves the high oxidation states of the transition metal cations in the cathodes, thus avoiding side reactions which reduce the oxidation state of the transition metals in the cathodes and avoiding the consequent detrimental structural changes that lead to degradation and capacity fading phenomena. We further employed XRD to investigate the suppression of Li/Ni cation mixing after cycling. In the FTOC‐SSE system, NCM811 retains a high I_003_/I_104_ ratio of 1.18 after 50 cycles, indicating minimal cation mixing. In contrast, the PEO‐Oxide system shows a lower ratio, suggesting more pronounced structural degradation.

XPS depth profiling of these cathodes after 10 cycles reveals pronounced fluctuations in the Ni 2p characteristic peaks on the surface of the cathodes cycled in the cells based on PEO‐oxide SSE, as shown in Figure 6n. Furthermore, with increasing etching depth, distinct signals associated with TM‐F bonds in the F 1s spectra become evident. In sharp contrast, the cathodes cycled in the cells based on FTOC‐SSE demonstrate stable Ni 2p signals as the etching depth increases (Figure 6m). The F1s peaks exhibit the appearance and progressive enhancement of favorable Li‐F signals, underscoring the FTOC‐SSE's superior stability.^[^ ^9^ ^]^ These findings demonstrate that Ni‐rich NCM cathodes indeed experience a structural collapse in contact with simple PEO composite solid electrolytes through side reactions that form TM‐F compounds along continuous decomposition of the SSE components.^[^ ^53^ ^]^ This process ultimately leads to the formation of thick surface films on the cathodes. In contrast, FTOC‐SSE exhibits intrinsic resistance to high‐voltage oxidative degradation. Li⁺‐EO coordination dominates the solvation structure in FTOC‐SSE, inducing the formation of a LiF‐rich CEI inner layer, effectively mitigating electrolyte decomposition and enhancing the entire cell's stability (anode, cathode, and electrolyte). All the above‐described spectral, microscopic, and electrochemical results are fully coherent, leading to the conclusions we reached. Compared to previous work, our design demonstrates significant advantages in relatively low lithium salt concentration, wide operating temperature range, and thin membranes in high voltage/high energy ASSLMBs (Figure 6l and Table S2, Supporting Information). These properties are intricately linked to practical applicability.

## 3 Conclusion

In summary, we have successfully used “fluoroetherized” titanium‐oxo clusters (FTOC), featuring fluoroether segments on the surface of nanocomposite components, as innovative prototypes to directly upgrade conventional poly(ethylene oxide) (PEO)‐based electrolytes into a new class of high‐voltage all‐solid‐state electrolytes. The fluoroether design and hybrid organic/inorganic nanostructures were validated as critical factors in enhancing electrochemical performance, owing to their superior composite compatibility and remarkable high‐voltage stability. The incorporation of FTOC reconstructs the Li⁺ solvation structure by introducing a weakly coordinating environment, which originates from the dilution of coordinating C–O–C segments within the PEO matrix. This strategy effectively promotes the formation of inorganic‐rich interfacial layers, facilitating excellent Li ions interfacial transport capability and leading to very uniform lithium metal plating and dissolution. The uniform Li transport processes induce superior uniform Li surfaces during prolonged periodic deposition/dissolution cycling. The FTOC‐based all‐solid‐state electrolytes exhibited enhanced mechanical robustness and exceptional interfacial compatibility. The uncompromised overall performance underscores the critical role of nanocomposite compatibility and the multifunctional design of fillers. Consequently, the optimized solid electrolytes demonstrate ultra‐stable cycling performance, sustaining over 9500 hours in symmetrical Li||Li cells and delivering outstanding stability for LiNi_0.8_Co_0.1_Mn_0.1_O_2_||Li full cells. Significantly, assembled LiMn_0.6_Fe_0.4_PO_4_||Li cells demonstrated an outstanding cycle life exceeding 1200 cycles, with no signs of degradation even at a cut‐off voltage of 4.5 V. Furthermore, we provided compelling evidence that FTOC effectively suppresses the progressive collapse of Ni/Mn structural sites in Ni‐rich NCM811 cathodes in contact with the PEO‐based electrolyte, demonstrating exceptional anti‐oxidation durability. This work not only expands the design library of multifunctional solid‐state electrolytes for next‐generation energy storage technologies but also introduces a new conceptual framework for developing other highly functional electrolyte systems.

## Conflict of Interest

The authors declare no conflict of interest.

## Supporting information



Supporting Information

## Data Availability

The data that support the findings of this study are available in the supplementary material of this article.
