# Supplementary material for: Fluoroether Design Enables High‐Voltage All‐Solid‐State Lithium Metal Batteries
Source: Adv Mater. 2025 Jul 1;37(36):2506020. doi: 10.1002/adma.202506020 (PMC12422082; doi:10.1002/adma.202506020)
Supplement: Supplementary file 1 — Supporting Information [file ADMA-37-2506020-s001.docx]

**Supporting Information**

**Fluoroether Design Enables High-Voltage All-Solid-State Lithium Metal Batteries**

*Yong Chen ^a^, Xu Yang ^a^, Tianyi Wang ^a^, Xiao Tang ^b^, Dongfang Li ^a^, Shijian Wang ^a^, Yaojie Lei ^a^, Yu Han ^c^, Shimou Chen *^, c^, Michel Armand ^d^, Doron Aurbach *^, e^, and Guoxiu Wang *^, a^*

[a] Y. Chen, X. Yang, D. Li, S. Wang, Y. Lei, Prof. G. Wang

Centre for Clean Energy Technology, University of Technology Sydney, Broadway, Sydney, NSW 2007, Australia

E-mail: Guoxiu.Wang@uts.edu.au

[[b] X. Tang

School of Chemistry and Chemical Engineering, Qingdao University, Qingdao, Shandong, 266071 China

[c] Y. Han, Prof. S. Chen

State Key Laboratory of Chemical Resource Engineering, Beijing Key Laboratory of Electrochemical Process and Technology of Materials, Beijing University of Chemical Technology, Beijing 100029, P.R. China

E-mail: [chensm@buct.edu.cn](mailto:chensm@buct.edu.cn)

[d] Prof. M. Armand

Centre for Cooperative Research on Alternative Energies (CIC EnergiGUNE) Basque Research and Technology Alliance (BRTA) Alava Technology Park Albert Einstein 48, 01510, Vitoria-Gasteiz, Spain

[e] Prof. D. Aurbach

Department of Chemistry, BINA-Bar-Ilan Institute of Nanotechnology and Advanced Materials and INIES – Israel National Institute for Energy Storage, Bar-Ilan University, Ramat-Gan, 5290002 Israel

E-mail: [doron.aurbach@biu.ac.il](mailto:doron.aurbach@biu.ac.il)

**Experimental Procedures**

**Synthesis of Materials:** For the synthesis of FTOC, microporous titanium-oxo clusters with a labile surface were initially prepared by employing a modified approach based on a previous report with modifications.^[1]^ Specifically, by combining ethylene glycol (EG, A.R.), Ti(OBu)_4_ (99%), and monocarboxylic acid (propionic acid, 98%), giant cyclic Ti-oxo clusters (TOC) with permanent porosity were self-assembled. After thoroughly washing away excess EG ligands with anhydrous tetrahydrofuran (THF, A.R.), 100 mg of overnight dried TOC was dispersed in 5 mL of dichloromethane (CH_2_Cl_2_, 99%). Following sonication, 0.5 mL long-chain fluoroether alcohol (F1EO, F(C_2_F_4_)_x_C_2_H_4_(C_2_H_4_O)_y_OH) ligand was added, and the solution was stirred overnight to ensure complete exchange with the long-chain fluoroether. A rapid addition of short-chain fluoroether alcohol ligand (F2EO, (CF_3_)_2_CHOH) was then introduced to facilitate rapid ligand exchange, thereby minimizing the remaining exchangeable EG ligands as much as possible and yielding precipitates (FTOC). The polyvinylidene fluoride-co-hexafluoropropylene (PVDF-HFP, M_w_~400000, 99%) framework was obtained through electrospinning technology, using a voltage of 20 kV and a flow rate of 0.5 mL/h to control film formation.

**Preparation of All-Solid-State Electrolytes:** To prepare all-solid-state PEO-SSEs, poly(ethylene) oxide (PEO, M_W_ = 600 000, 99%) and LiTFSI (99.9%) were dissolved in anhydrous acetonitrile (CH_3_CN, 99.9%). The EO to Li molar ratio was 16:1. The mixture solution was continuously stirred overnight to ensure the formation of a homogeneous solution. After resting for 1 h, the solution was cast onto the PVDF-HFP framework, with the framework thickness used to control the resulting film thickness. After drying at room temperature for over 8 h, the samples were transferred to a vacuum drying oven at 60 °C for 48 h to ensure complete solvent removal. High-salt-concentration PEO-based SSEs were prepared with an EO to Li molar ratio of 8:1, denoted as PEO_8_-SSEs. FTOC-SSEs were prepared with a facile two-step process. First, after ligand exchange, the obtained FTOC was ultrasonically dispersed in CH_3_CN for at least 15 minutes to form a homogeneous FTOC dispersion. Then, the specified FTOC amount was slowly added to the as-prepared PEO-LiTFSI acetonitrile solution. The obtained solution was stirred overnight. After resting for 1 h, the solution was cast onto the PVDF-HFP framework. The FTOC-SSEs underwent the same drying process as the regular PEO-SSE films. PEO-oxide SSEs were prepared following the same protocol as FTOC-SSEs, substituting FTOC with an equivalent amount of oxide nanoparticles (TiO_2_ < 25 nm; Al_2_O_3_ ≤ 50 nm). As a comparative experiment, the unsupported FTOC-SSE was fabricated via a casting method, resulting in a membrane thickness of approximately 150 μm.

**Materials Characterization:** The surface and cross-sectional morphologies of PEO-based SSEs were observed using a Zeiss Evo LS15 SEM and a field-emission Zeiss Supra 55VP SEM. The elemental analysis and mappings were captured by a Bruker SDD XFlash 5030 detector. X-ray diffraction (XRD) patterns were collected to analyze the crystal structure on a Bruker D8 Discover XRD with the scanning range from 10° to 80° and Cu-Kα radiation. The thermostability of the prepared SSEs was analyzed by SDT-Q600 under N_2_ atmosphere from 25~600 ℃ at 10 °C min^−1^. Bruker Advance 400 NMR was used to characterize the field shift of ^7^Li with/without FTOC in PEO electrolytes. Nicolet FT-IR 6700 was used to perform Fourier-transform infrared spectroscopy (FT-IR). The scanning range was set from 3600 to 400 cm^−1^ with a scanning resolution of 4 cm^−1^ and 64 scans per sample. Renishaw Raman spectroscopy was used here. DSC was tested using a Q1000 Modulated Differential Scanning Calorimeter (TA Instruments) under flowing N_2_ at 10 ℃ min^−1^. The morphologies of electrodes were observed by transmission electron microscopy (TEM, JEM−F200, Japan), and cryogenic transmission electron microscopy (Cryo-TEM) was used to test the SEI on the Cu current collected by XL-30 ESEM (Philips) with K1250X (Quorum) as a low-temperature cryogenic transfer system. Time-of-flight secondary ion mass spectrometer (ToF-SIMS) analysis was conducted by the M6, IONTOF (Germany). Cs^+^ sputtered at 1 keV, analysis in negative polarity by Bi^+^ of 30 kV. Atomic Force Microscopy (AFM, Park XE7) was used to test the morphologies of SSEs. X-ray photoelectron spectroscopy (XPS) was performed by a Kratos AXIS Supra photoelectron spectrometer. All peaks were calibrated based on the C 1s peak (284.8 eV). For the analysis of solid electrolyte interphase (SEI) by XPS, the cycled electrodes were transferred into an argon-filled transfer chamber (Thermo Fisher K-Alpha + XPS). Synchrotron-based infrared mapping was collected at the Australian National Synchrotron Radiation Research Centre. X-ray absorption fine structure spectra (XAS) were collected at the Australian National Synchrotron Radiation Research Centre and analyzed using the ATHENA software package.^[2]^

**Electrochemical Characterizations:** Biologic VMP-300 potentiostats were used for electrochemical impedance spectroscopy (EIS), chronoamperometry, and linear sweep voltammetry (LSV). To measure the ionic conductivity of SSEs at different temperatures, the SSE film was sandwiched between stainless steel (SS) disks in a SS|SSE|SS configuration. The ionic conductivity, σ, was calculated using

$\sigma=\frac{d}{RS}$ (1)

where *d* is the thickness of the SSE, *S* is the contact area between SSE and SS, and *R* is the resistance measured from EIS in the frequency range of 1 MHz-0.1 Hz. LSV analysis was conducted between 2.8 and 6.0 V at a scan rate of 0.1 mV s^−1^ with Li||SS asymmetric cells to measure the electrochemical window. Electrochemical floating experiments were measured with NCM811||Li full cells, which were charged to 4.0 V first, then held at progressively higher voltages, each for 10 h. To demonstrate the improved ionic conductivity, all battery tests were conducted at 50 °C unless otherwise specified, which is lower than the conventional operating temperature typically adopted in similar studies. To evaluate electrochemical stability, CR2032-type coin cells were used to assemble Li metal batteries for electrochemical performance evaluation. The battery cycling performance was measured with multichannel Neware or LANHE battery testers. The cycling stability and rate capability of Li||Li, Li||LFP, Li||LMFP, and Li||NMC811 batteries were evaluated using the same battery testing systems.

**Fabrication of Cells**

Symmetrical lithium metal batteries were assembled to evaluate the compatibility between SSEs and Li metal anodes. The SSE films were sandwiched between Li metal disks in a Li|SSE|Li cell configuration. To mitigate rapid voltage polarization due to insufficient lithium-ion supply under high current densities, an additional FTOC-SSE with high lithium salt concentration (the EO to Li molar ratio was 10:1) was employed for interfacial modification, with the membrane thickness controlled to less than 50 µm. To use current battery fabrication techniques, conventional methods were employed for cathode preparation. The LFP cathodes were prepared by mixing LFP powder, conductive carbon black (Super P, Aladdin), polyvinylidene difluoride (PVDF, Aladdin) binder at a weight ratio of 8:1:1 in N-methyl-2-pyrrolidone (NMP, 99.9%) solvent to form a smooth slurry. The slurry was then magnetically stirred for 12 h and cast onto carbon-coated Al. After drying at 80 °C in a vacuum oven overnight and removing the NMP solvent, the LFP-based cathode layer was punched into disks, with a common mass loading of over 2.0 mg cm^−2^. The interfacial treatment solution was prepared by adding 10 g of CH_3_CN, 0.1 g of succinonitrile, and 0.1 g of LiDFOB to the previously obtained FTOC-SSE solution. After thoroughly mixing the solution to obtain a homogeneous mixture, an appropriate amount was cast onto the porous cathode surface. The samples were then dried under vacuum at 60 °C for 48 h, followed by drying at 80 °C for 2 h, ensuring complete removal of any residual solvent. Similarly, other cathodes, including LMFP, and NCM811 were prepared by the same procedure. The cathode slurry compositions for PEO-SSE and PEO-oxide-SSE were identical to that of FTOC-SSE, except that no FTOC was added. The battery fabrication processes were performed inside a glove box ([O_2_] < 0.1 ppm, [H_2_O] < 0.1 ppm) filled with ultrapure Ar (≥99.999%).

**Computational Methods**

DFT: The DFT-D3 calculation was carried out using Gaussian 16 software.^[3]^ The structural optimization was performed at M06-2X/def2-TZVP level, which has been proved to be reliable in identifying molecular interactions.^[4,5]^ Vibrational frequencies were calculated at the same level of theory to ensure that the optimized structures have no imaginary frequency as the global minima. The Gibbs free energy changes (Δ*G*) were calculated by:

Δ*G* = *G*_P_ - *G*_R_ (2)

where ‘*G*_R_’ is the total Gibbs free energy of reactants and ‘*G*_P_’ is the total Gibbs free energy of products. The Gibbs free energies were the correction of single-point energies, which were calculated at M06-2X/def2-TZVP level. The generalized gradient approximation (GGA) method with Perdew-Burke-Eumzeahof (PBE) function was employed to describe the interactions between the core and electrons. The force and energy convergence criterion were set to 0.002 Ha Å^−1^ and 10^−5^ Ha, respectively. The binding energy (*∆E*) was calculated using formula:

∆(𝐸) = 𝐸_total_ − 𝐸_1_ − 𝐸_2_ (3)

where the *E*_total_ is the energy of the optimized system, *E*_1_ is the energy of the material, *E*_2_ is the energy of Li^+^.

COMSOL: COMSOL Multiphysics was used to study the Li^+^ diffusion model in the FTOC-SSEs and PEO-oxide SSEs. According to the characteristics of the simulation model, a simplified 2D method is used for the Li^+^ diffusion model. The simulation model accords with formula:

∇(−𝐷∇𝑐) = 0 (4)

Where *c* and *D* are concentration (mol m^−3^) and diffusion coefficient, respectively. The diffusion coefficients are 5 × 10^−10^ m^2^ s^−1^, 1 × 10^−10^ m^2^ s ^−1^ and 5 × 10^−9^ m^2^ s^−1^ for FTOC, PEO matrix and FTOC-SSE, respectively. The Li^+^ transport within FTOC nanoparticles, based on Fick’s law, was simulated using the finite element software COMSOL Multiphysics 5.5 (governing equation shown in Eqs. 1 and 2). TiO_2_ and FTOC nanoparticles were separately modeled in two distinct geometries, each with a computational domain of 20 nm in side length.

$\nabla$J$+u\nabla_{c}=R$ (5)

J$=-D_{eff}\nabla_{c}$ (6)

where **J** is Li^+^ ion flux, **u** is the flow rate, *c* is Li^+^ concentration, *R* is the reaction term, $D_{eff}$ is the diffusion coefficient depending on the material properties of each domain. The values of **u** and *R* were zero throughout the simulations.

MD: The MD was performed using the large-scale atomic/molecular massively parallel simulator (LAMMPS) in a simulation box of 120 Å ×120 Å × 120 Å. The content of each component is precisely according to the molar ratio of the electrolyte membrane. The content of each substance is consistent with the molar ratio of the experimental process. The bonded, non-bonded interactions and the ionic charges of the PEO chains and the TFSI^−^ anions are, respectively, described by the Polymer Consistent Force field (PCFF) all-atoms force field. The non-bonded interactions between any atomic pair are calculated by the Lorentz-Berthelot combination rules. Given that all the atoms in the system are carrying a charge, the electrostatic interaction is described by the Coulomb potential with a cutoff radius of 10.0 Å. The long-range Coulombic interactions were solved using the Particle-Particle Particle-Mesh algorithm with an accuracy value of 10^−4^. For each simulation, energy minimization was first employed to relax the simulation box. Then, an NPT ensemble with a 1.0 fs time step was employed to optimize the simulation box, where the temperature was set to 500 K and 300 K, and the pressure was set to 1 atmosphere. The NPT optimization time was set to 0.5 ns at 500 K and 0.5 ns at 300 K, which is long enough to obtain a stable box size. During the relaxation process, another 1.0 ns NVT simulation was performed to obtain RDF and MSD results. In all the MD simulations, the motion of atoms was described by classical Newton’s equations, which were solved using the Velocity-Verlet algorithm.


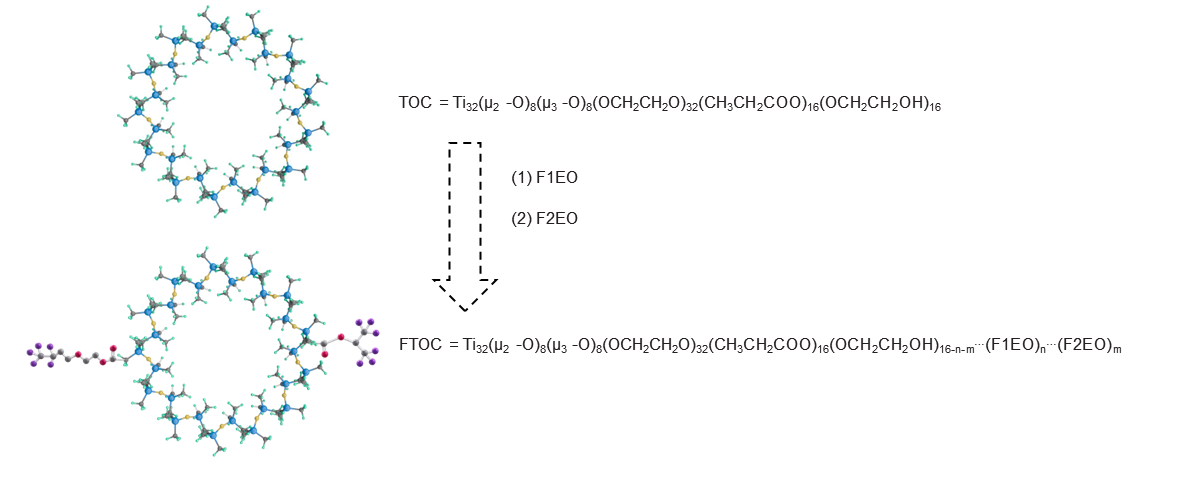


**Figure S1**. Schematic of the ligand substitution process from TOC to FTOC.

**Figure S2**. (a) ESP of F1EO (F1EO is a deprotonated fluoroether alcohol, considering its specific coordination environment. It is simplified with x = 1 and y = 1). The binding energy between Li^+^ and PEO (b), and Li^+^ with FTOC (c).


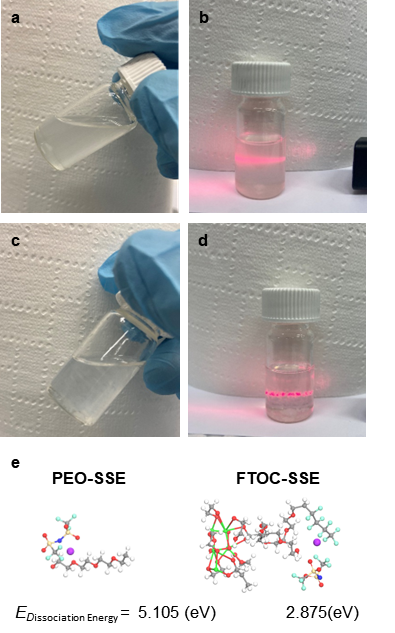


**Figure S3**. The dissolution ability of LiTFSI. Anhydrous chloroform was used to verify the lithium salt-dissolving capability of FTOC. a-b) In the FTOC system, a stable turbid phase was observed, with only a minor amount of undissolved LiTFSI settling at the bottom. In contrast, a-b) the TOC system exhibited pronounced agglomeration and significant sedimentation. (e) The E _Dissociation energy_ of LiTFSI in PEO-SSE and FTOC-SSE.


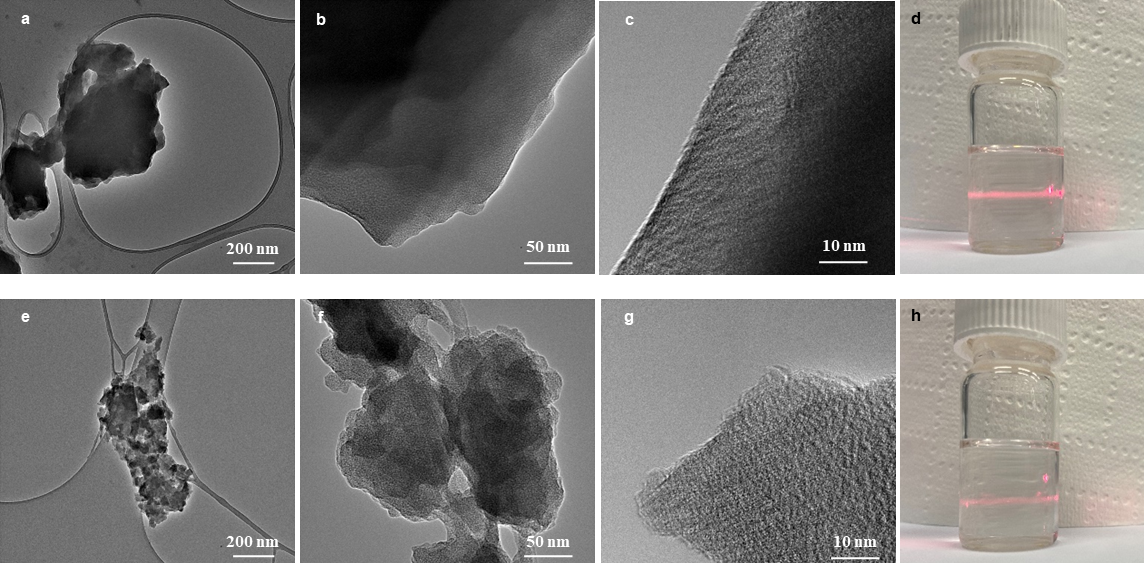


**Figure S4**. TEM images and their Tyndall effect of TOC (a-d) and FTOC (e-h), indicating their reduced particle size and excellent nanoparticle dispersion after the introduction of the fluoroether.


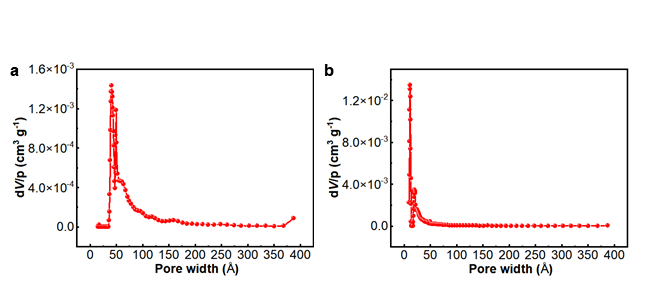


**Figure S5**. N_2_ adsorption/desorption isotherms of TOC (a) and FTOC (b). The abundant ethylene glycol ligands on the TOC surface enhance the interactions between individual TOC particles, facilitating their self-assembly into large mesoporous structures. Following ligand exchange, the introduction of fluoroether functional groups on the surface promotes the uniform dispersion of FTOC, resulting in finely dispersed particles.


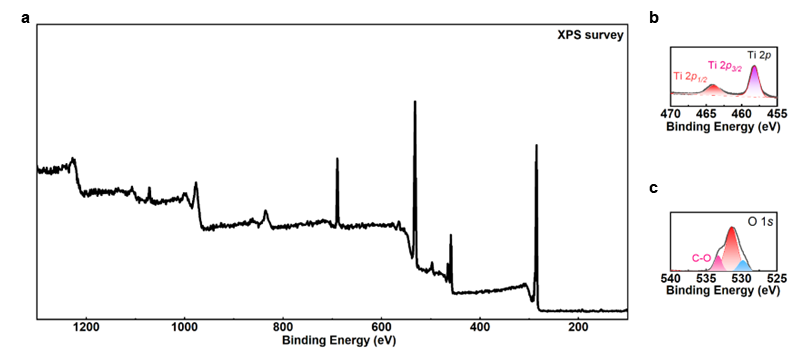


**Figure S6**. XPS analysis of TOC and FTOC, including (a) survey spectra, (b) Ti *2p* spectra, and (c) O *1s* spectra.

The XPS Ti *2p* and O *1s* spectra provide clear evidence for the formation of Ti–O clusters.


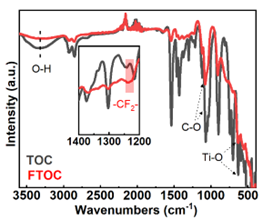


**Figure S7**. FTIR spectra of FTOC and TOC nanoparticles.


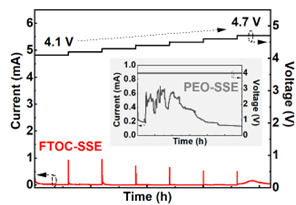


**Figure S8**. Electrochemical floating tests of NCM811||Li cells (the inset for PEO-SSE case).


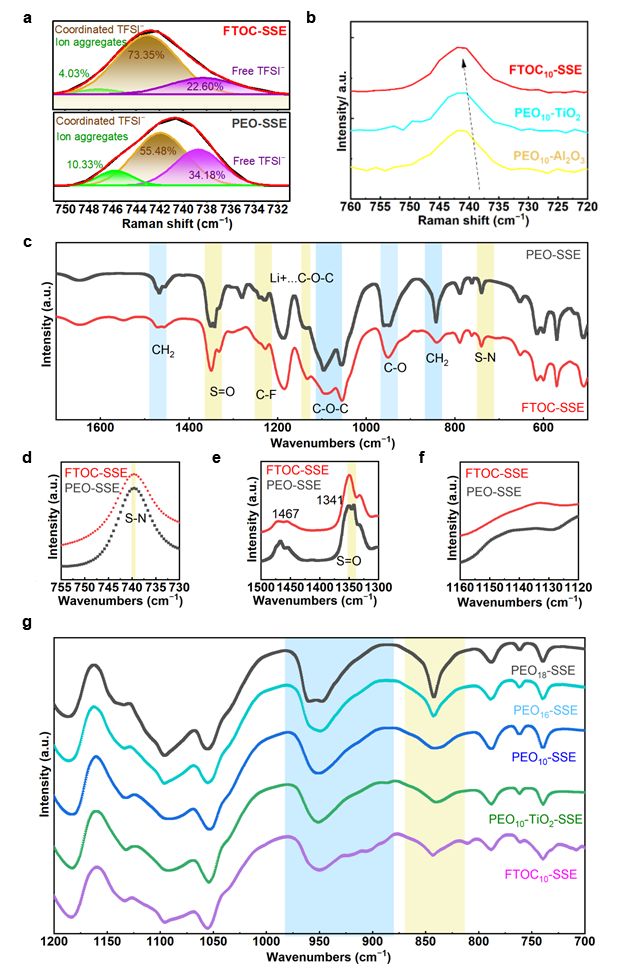


**Figure S9**. Raman and FT-IR spectra of PEO-based SSE and FTOC-based SSE. (a) Raman spectra results (720-755 cm^−1^) of PEO-based SSE and FTOC-SSE. (b) Raman spectra results (720-755 cm^−1^) of PEO-based SSE and FTOC-SSE with different fillers ( the same Li-salt concentration, using EO:Li^+^=10). (c-f) FT-IR spectra of PEO-based SSE and FTOC-SSE.^[6]^ (g) FT-IR spectra of PEO-based SSE and FTOC-SSE with different Li-salt concentrations.

Quantitative analysis in Figure S9a reveals that the incorporation of FTOC effectively promotes the dissociation of LiTFSI in the electrolyte. Furthermore, Figure S9b compares the influence of conventional oxide nanofillers (TiO_2_, and Al_2_O_3_) on LiTFSI dissociation and Li⁺ solvation under identical salt concentrations. The pronounced blue shift in FTOC-SSE indicates that the introduction of FTOC induces a weaker solvation environment of Li⁺ in the electrolyte species.

Figure S9c confirms the significant impact of FTOC incorporation on the PEO-LiTFSI matrix, as revealed by FTIR spectra (1175-700 cm⁻¹). The sharp CH_2_ rocking peak at 844 cm⁻¹ in PEO-SSE becomes broader with FTOC addition, indicating increased EO chain torsion and segmental relaxation. Figures S9d and S9e confirm an increased coordination ratio between PEO and TFSI⁻, accompanied by broadened peaks, indicating that the strong Lewis acidity of FTOC interacts with anions and competes with PEO chains for TFSI⁻ coordination. This competition weakens the interaction between PEO and TFSI⁻ anions, which is consistent with the reduced binding energy observed in our calculations. Furthermore, Figure S9f provides direct evidence for the weakened coordination between Li⁺ and EO segments.^[7–9]^

A detailed comparison with PEO and TiO_2_ (Figure S9g) shows that increasing LiTFSI content leads to progressive peak broadening, reflecting enhanced amorphous character in the PEO-SSE. Under high salt conditions, distinct C–O–C stretching bands at 1060, 1100, and 1108 cm⁻¹ correspond to crystalline, Li⁺-coordinated, and amorphous regions, respectively. The red shift and broadening of the 964 cm⁻¹ C–O peak further confirm FTOC-induced structural relaxation. Therefore, FTOC not only acts as a plasticizer but also participates in Li⁺ coordination, consistent with the binding energy calculations.


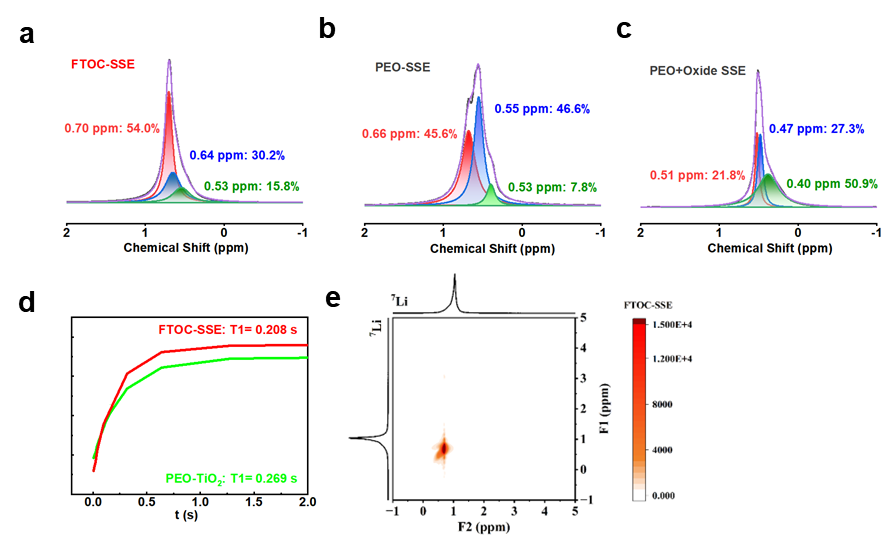


**Figure S10**. (a-c) ^7^Li ss-NMR spectrum of FTOC-SSE,PEO-SSE, and PEO-Oxide SSE. (d) T1 results for FTOC-SSE and PEO-Oxide SSE. (e) 2D ^7^Li-^7^Li exchange spectra.

Solid-state NMR reveals three peaks: the high peak corresponds to weakly coordinated Li⁺ (Li⁺-EO), while the intermediate and lower peaks arise from ion pairs. The upfield shift in the ^7^Li solid-state NMR spectrum suggests a reduced interaction between Li⁺ and coordinating species. FTOC incorporation increases the intensity of the 0.70 ppm peak, indicating a dominant weak coordination environment (~54%). In contrast, oxide fillers reduce this signal.


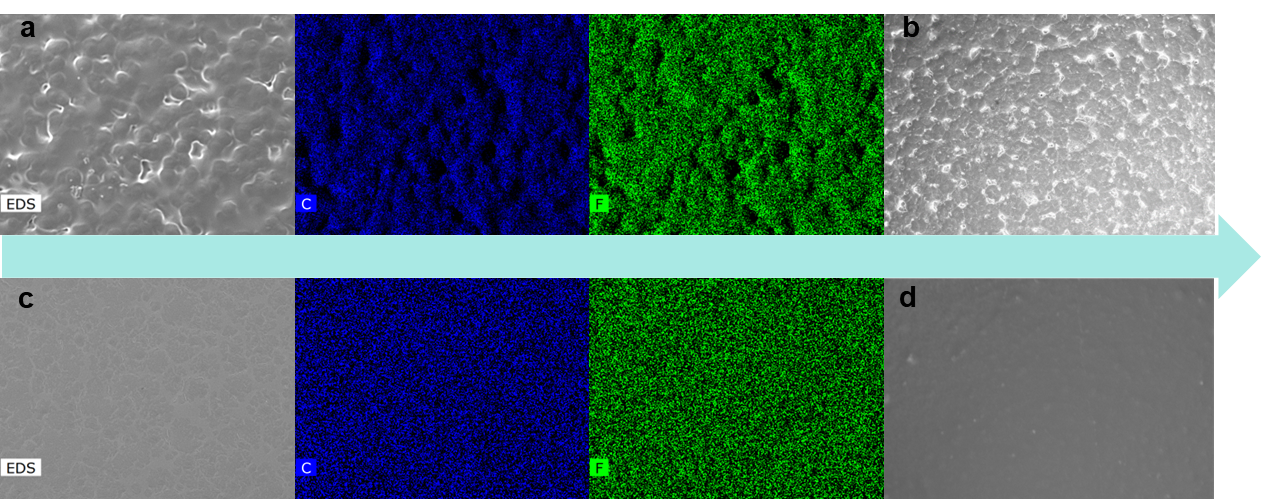


**Figure S11**. SEM and EDS mappings for PEO-SSE (the upper) and FTOC-SSE (the bottom).


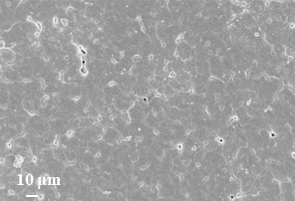


**Figure S12**. A SEM image of the surface morphology for PEO-oxide SSE.


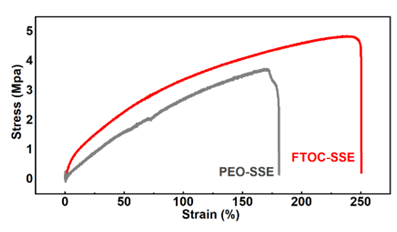


**Figure S13**. Stress-strain curve of FTOC-SSE and PEO-SSE membranes.


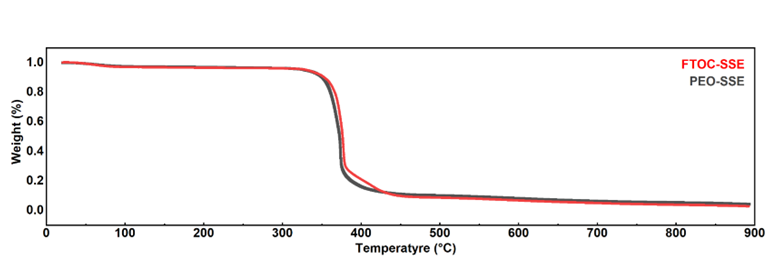


**Figure S14**. TGA results of FTOC-SSE and PEO-SSE membranes.


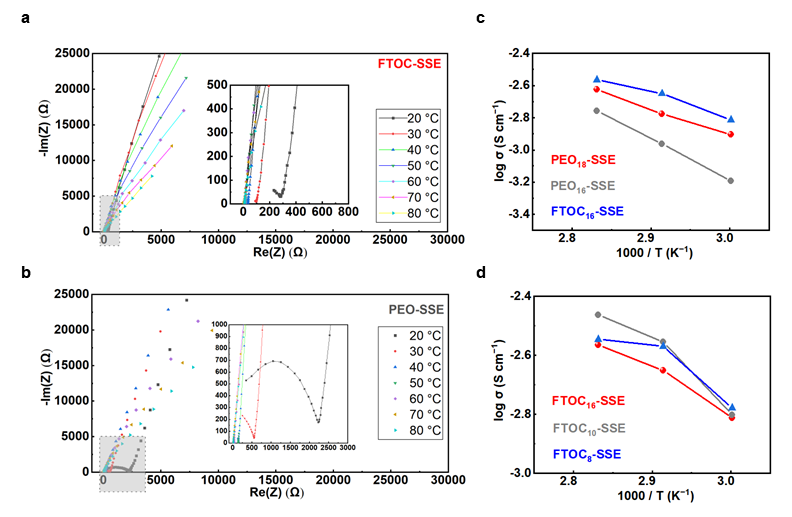


**Figure S15.** The ionic conductivity test for SS||SS symmetric cells. a) EIS Nyquist plots of FTOC-SSE, b) EIS Nyquist plots of PEO-SSE from 20 °Ϲ to 80 °Ϲ. (c) The ionic conductivities of PEO-SSEs in the temperature range from 60 °C to 80 °C. (d) The ionic conductivities of FTOC-SSEs in the temperature range from 60 °C to 80 °C.


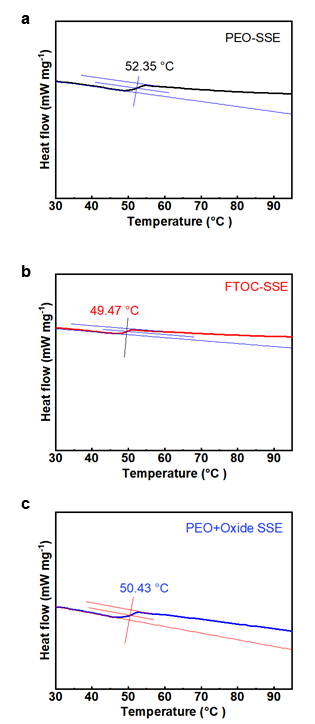


**Figure S16**. Results of DSC measurements and the T_m_ identification of SSEs as indicated.

The pure PEO-SSE electrolyte shows the highest Tₘ, while the addition of oxides reduces Tₘ by disrupting PEO crystallinity. The FTOC-SSE exhibits a further decrease in Tₘ due to the organic-inorganic hybrid structure.

**Figure S17**. XRD results of FTOC-SSE and PEO_16_-SSE films.


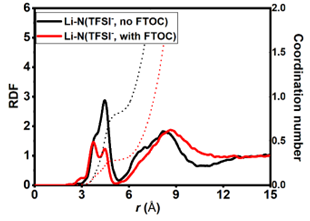


**Figure S18**. Calculated RDF of Li^+^-N in the TFSI^−^ anions.


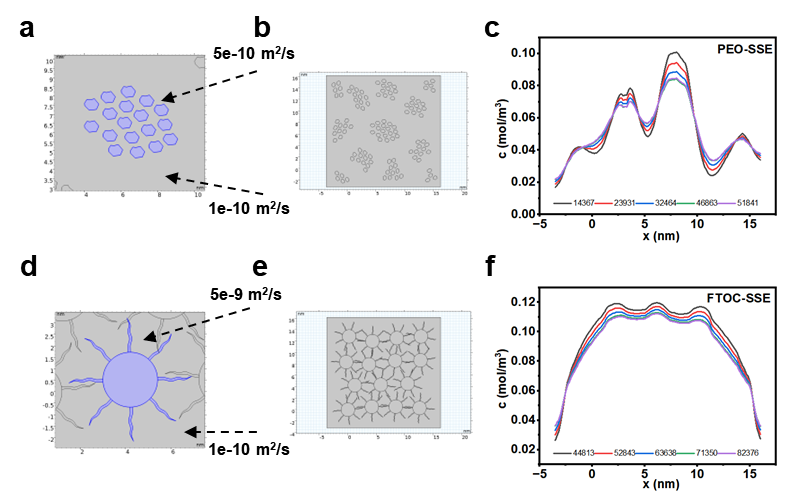


**Figure S19**. a-b) COMSOL results for diffusion coefficient in PEO-Oxide SSE, and c) the related mesh independence verification. d-e) COMSOL results for diffusion coefficient in FTOC-SSE, and f) the related mesh independence verification.

To ensure the reliability of the simulation, a mesh independence verification was performed, as shown in Figure S19f and i. The computational domain was defined as 20 × 20 nm, with an initial Li⁺ concentration of 0.5 mol m⁻^3^, and a total simulation time of 10 ns. For the FTOC nanoparticles, the maximum relative deviation was 1.05% when the mesh numbers were 71,350 and 82,376. For the TiO_2_ nanoparticles, the maximum relative deviation was 1.25% when the mesh sizes were 46,863 and 51,841.^[10,11]^


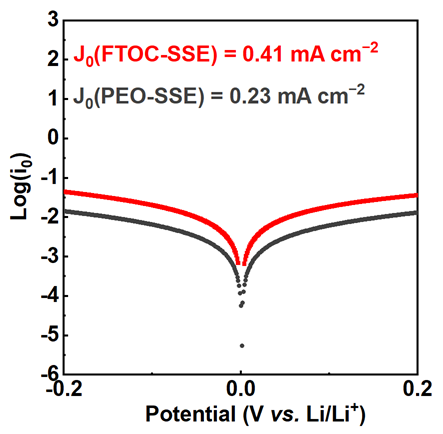


**Figure S20**. Potentiodynamic behavior of Li deposition/dissolution processes in symmetric Li cells with PEO-SSE and FTOC-SSE as indicated. Tafel plots were extracted from Log(i_0_) *vs.* potential (V) curves.


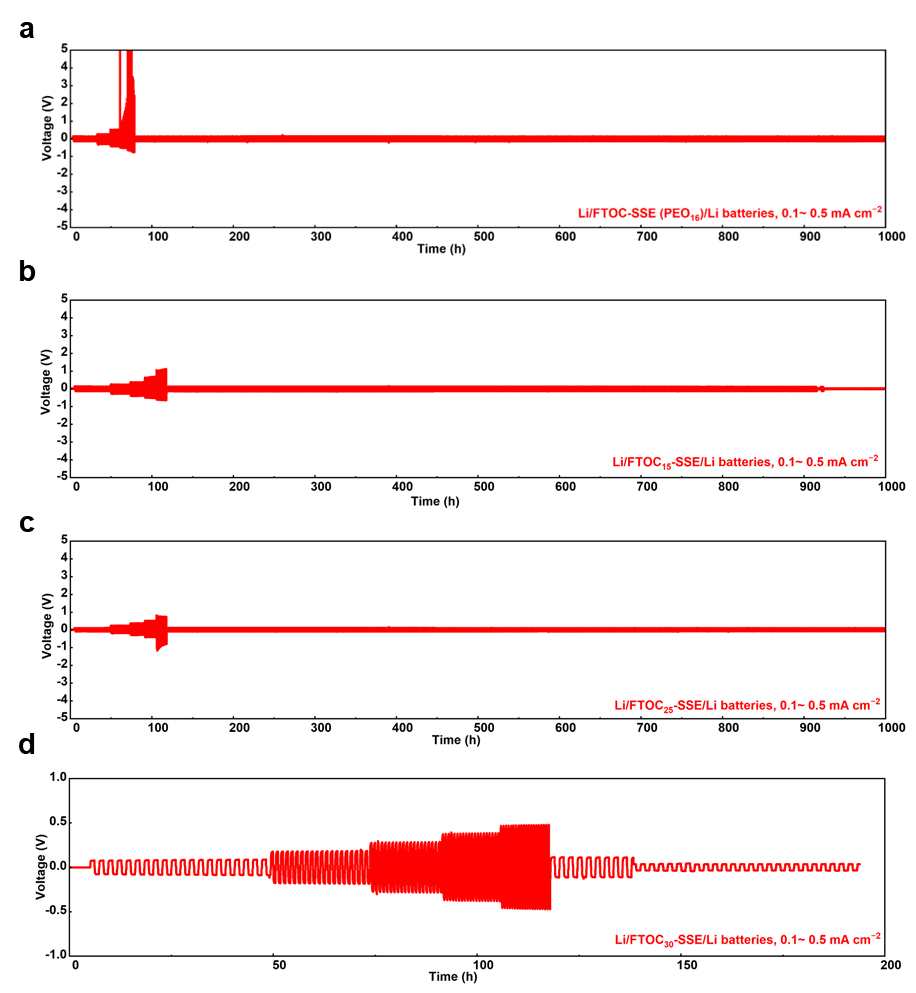


**Figure S21**. Rate capability testing of symmetric Li cells, which is used for rapid screening of optimized components. a) Rate performance and long cycling performance of Li|FTOC-SSE (PEO_16_)|Li cells. The EO to Li molar ratio was 1:16. b) Rate performance and long cycling performance of a typical Li|FTOC_10_-SSE|Li cell. The loading of FTOC was 10 wt.%. c) Rate performance and long cycling performance of a typical Li|FTOC_25_-SSE|Li cell. The loading of FTOC was 25 wt.%. d) Rate performance and long cycling performance of Li|FTOC_30_-SSE|Li cell. The loading of FTOC was 30 wt.%.

The rate performance and cycling stability of the Li|FTOC-SSE (PEO_16_)|Li cells show significant polarization from 0.4 mA cm^−2^, primarily due to Li^+^ depletion. Therefore, in the subsequent lithium battery tests, an ultrahigh-salt-content PEO matrix with an EO to Li molar ratio of 10:1 was introduced at the interface to improve interfacial stability. The overall membrane thickness was maintained below 50 µm. From (b-d), Li||Li cells demonstrated that incorporating FTOC effectively reduces voltage polarization. However, an excessive amount of FTOC, despite further reducing voltage polarization, may induce soft short-circuits in the cells, likely due to the increased Ti content in the FTOC moieties.


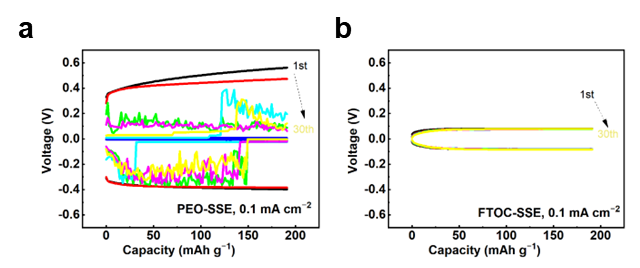


**Figure S22**. Voltage-capacity curves of Li|PEO-SSE|Li cell a) and Li|FTOC-SSE|Li cell b).

**
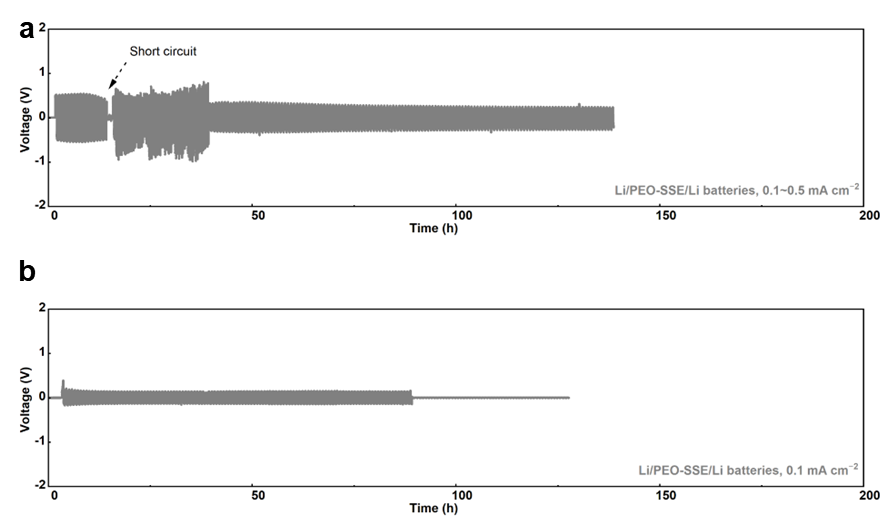
**

**Figure S23**. Rate performance a) and plating/stripping cycling performance at 60 °C, and b) of symmetric Li|PEO-SSE|Li cells at 60 °C.

PEO-SSE demonstrates rapid internal short-circuiting and unstable voltage fluctuations with the increase of current density. Under low current density conditions, PEO-SSE remains highly susceptible to short-circuiting.


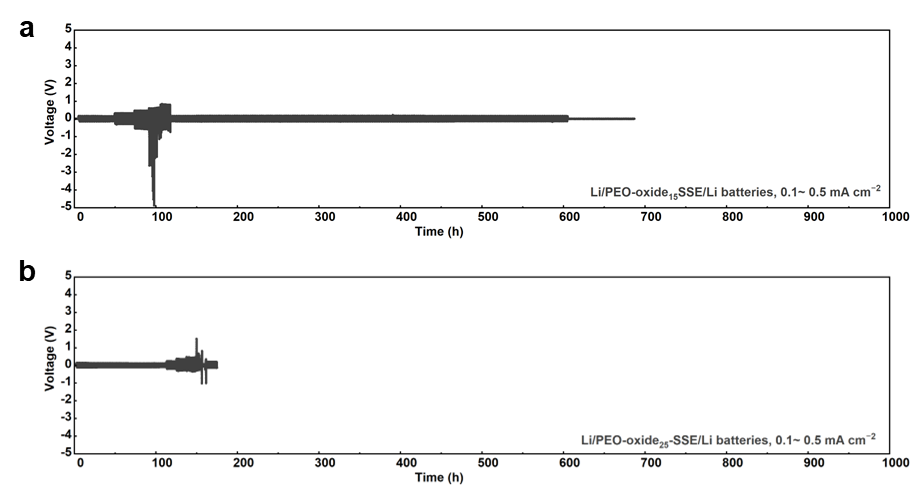


**Figure S24**. Rate performance plating/stripping cycling performance of a) symmetric Li|PEO-oxide_15_ SSE|Li cells at 60 °C, and b) symmetric Li|PEO-oxide_25_ SSE|Li cells at 60 °C.


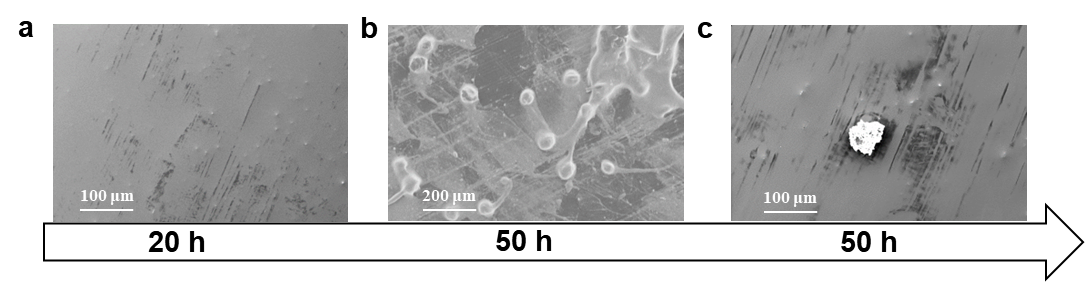


**Figure S25**. Lithium metal deposition of PEO-SSE at 60 °C characterized by SEM (harvested from cells after cycling).


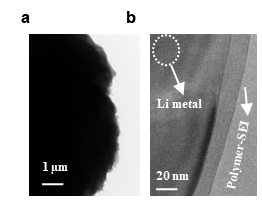


**Figure S26**. Cryo-TEM images of a typical Li anode cycled in a cell containing PEO-SSE.


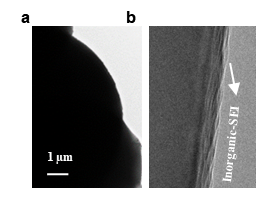


**Figure S27**. Cryo-TEM images of a typical Li anode cycled in a cell containing FTOC-SSE.


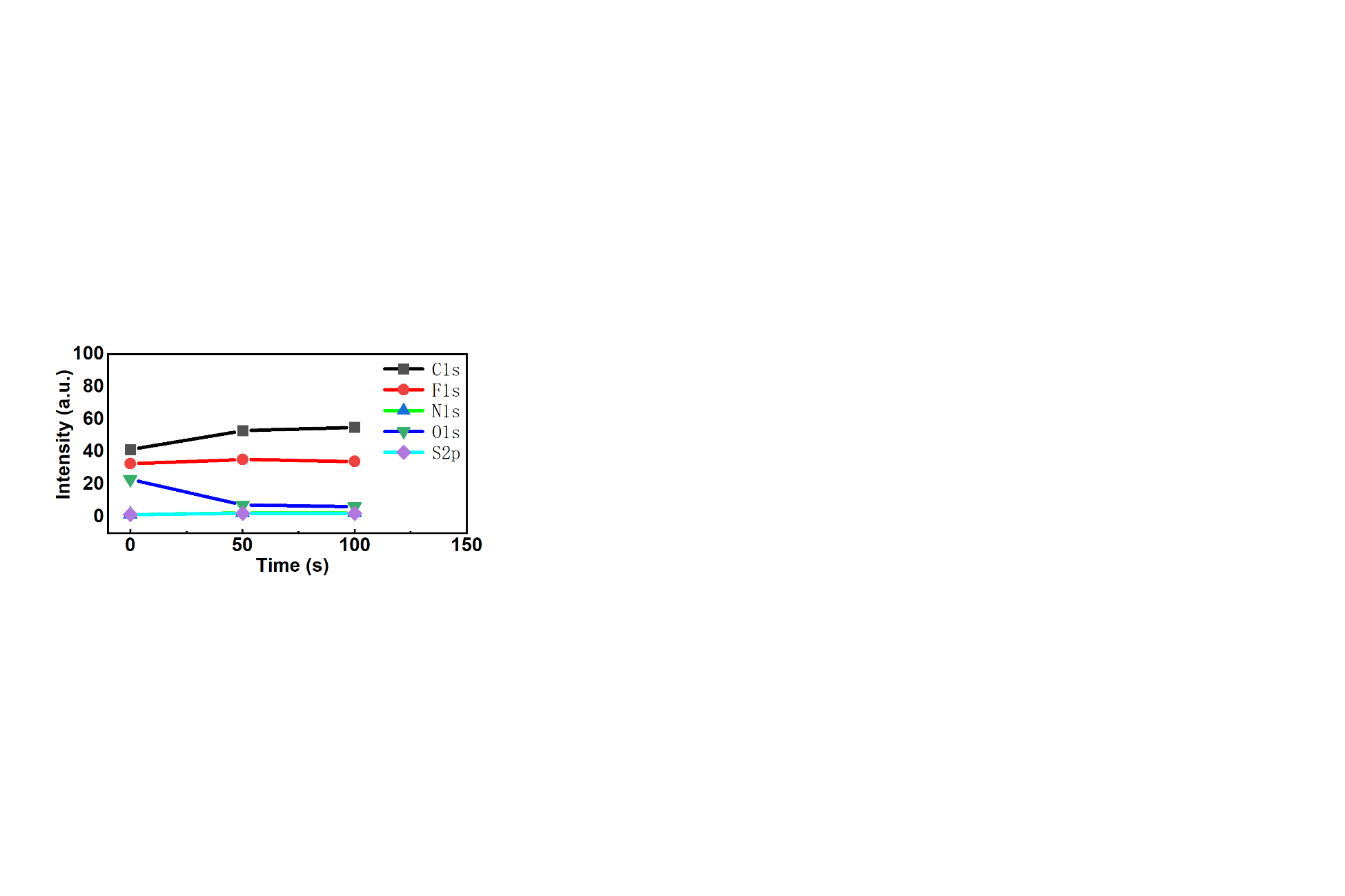


**Figure S28**. Elemental composition variation with etch time related to a typical Li anode after being cycled in a Li|FTOC-SSE|Li cell, measured by XPS during sputtering.


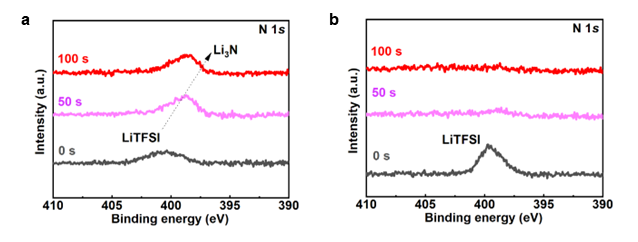


**Figure S29**. N 1s X-ray photoelectron spectra (XPS) of typical Li anodes after being cycled in cells containing a) FTOC-SSE and b) PEO-SSE.


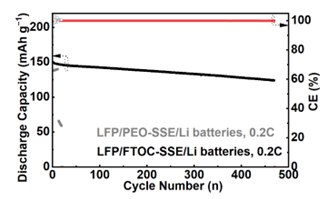


**Figure S30**. Results of cycling stability experiments of Li||LFP cells containing the two types of solid electrolytes as indicated at 60 °C and 0.2C (constant current experiment, 1C = 170 mAh g^−1^).


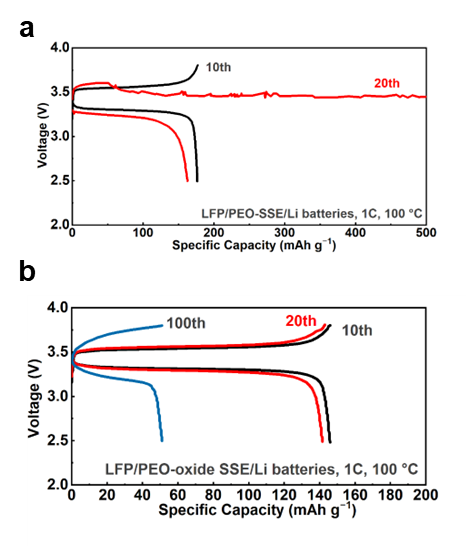


**Figure S31**. Charge and discharge voltage profile curves during galvanostatic cycling at 100 °C and 1C (constant current experiment, 1C = 170 mAh g^−1^) of Li||LFP cells containing. a) PEO-SSE and d) PEO-oxide SSE.


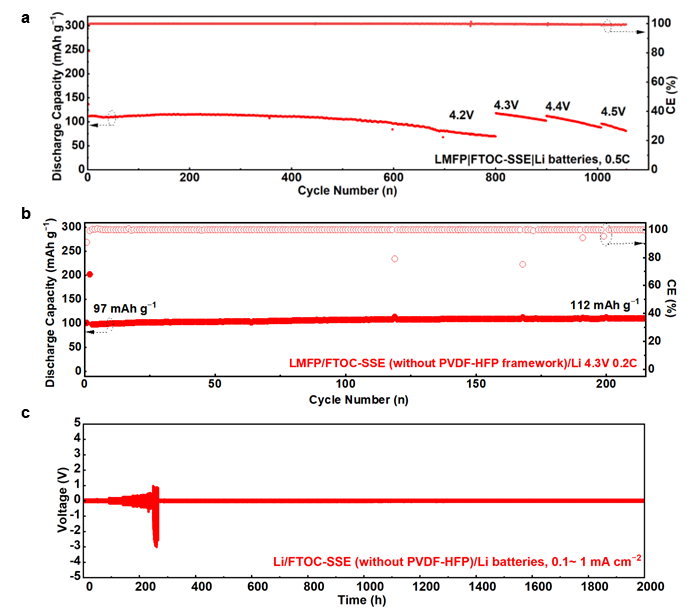


**Figure S32.** a) Results of galvanostatic cycling experiments of Li||LMFP cells containing FTOC-based SSE, at 0.5C rate (1C = 170 mAh g^−1^) and 50 °C. b) Li||LMFP cells containing FTOC-SSE (without PVDF-HFP and no CEI additives), at 0.2C rate (1C = 170 mAh g^−1^) and 50 °C. c) Rate performance of Li||Li cell using FTOC-SSE (without PVDF-HFP) at 60 °C.


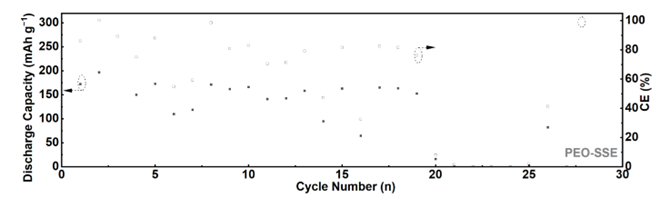


**Figure S33.** Results of galvanostatic cycling performance tests of Li|PEO-SSE|NCM811 batteries at 60 °C and 0.2C (constant current experiment, 1C = 200 mAh g^−1^).


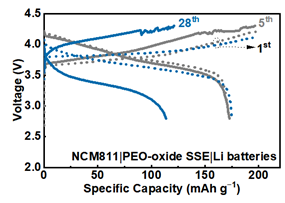


**Figure S34.** Charging and discharging voltage profiles of Li|PEO-oxide SSE|NCM811 batteries at 60 °C and 0.2C (constant current experiment, 1C = 200 mAh g^−1^).


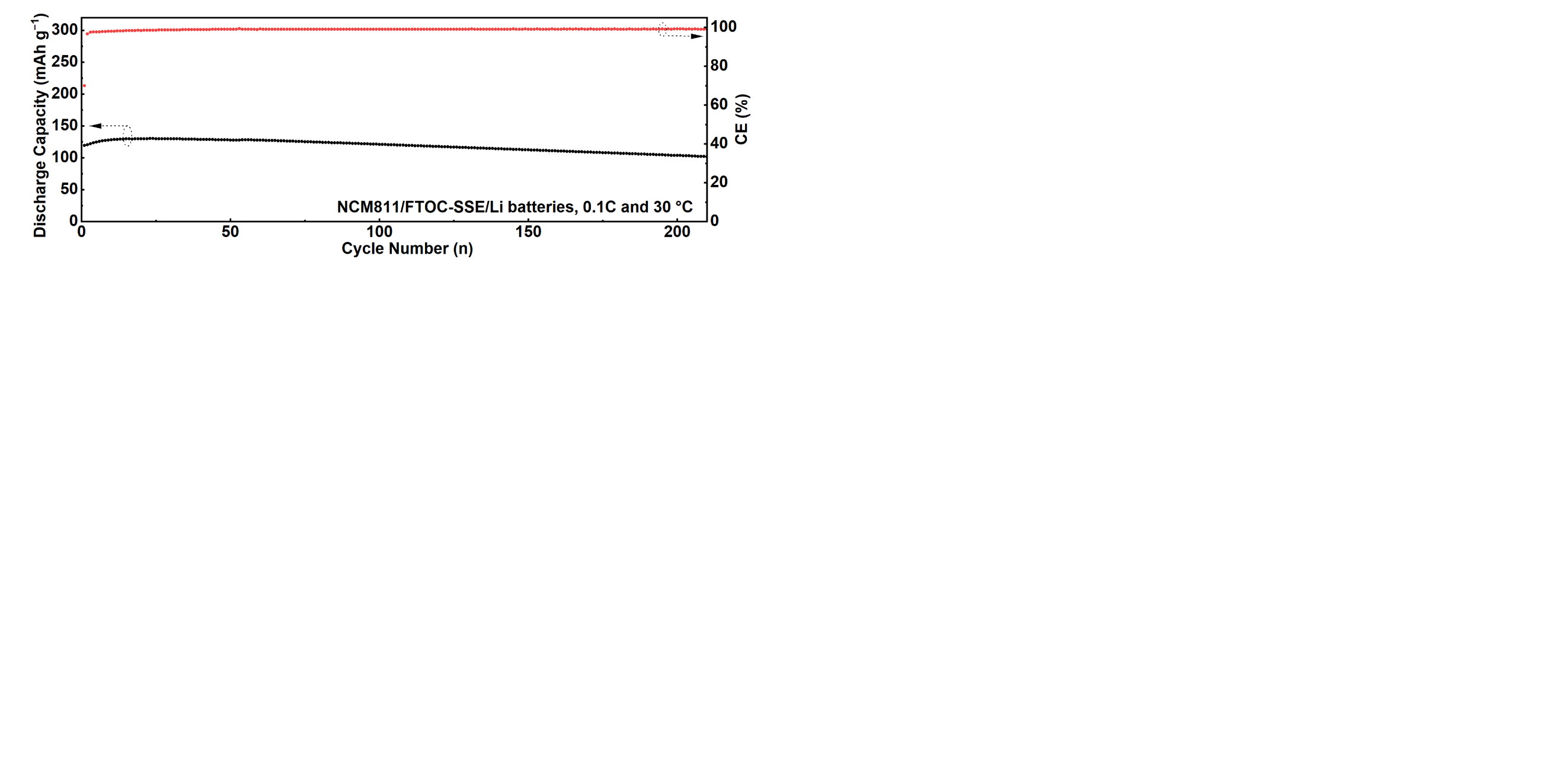


**Figure S35.** Results of galvanostatic cycling performance tests of Li|FTOC-SSE|NCM811 batteries at 30 °C and 0.1C (constant current experiment, 1C = 200 mAh g^−1^).


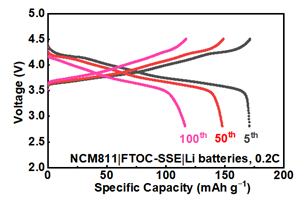


**Figure S36.** Charging and discharging voltage profile curves, constant current operation of Li|FTOC-SSE| NCM811 batteries, charged up to 4.5V.


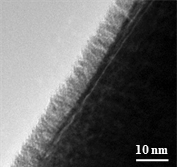


**Figure S37.** A TEM image of the NCM811 electrode (cross-section observation) taken from a Li|FTOC-SSE| NCM811 cell after 50 cycles.


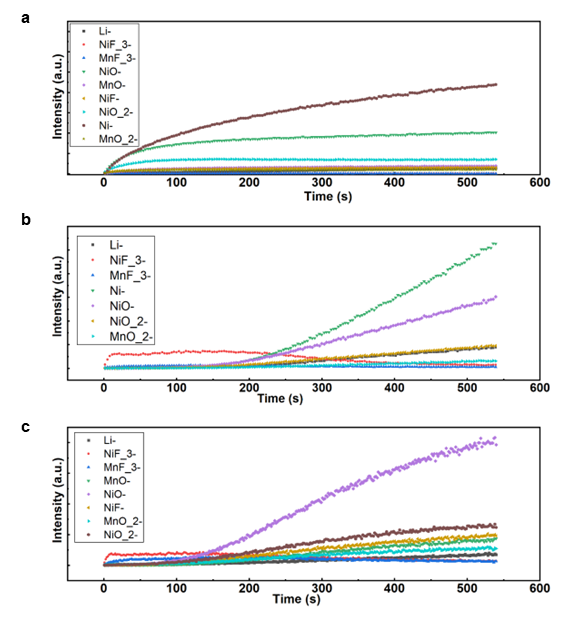


**Figure S38.** TOF-SIMS results of NCM811 electrodes taken from a) Li|FTOC-SSE| NCM811 cell after 10 cycles. b) Li|PEO-oxide SSE|NCM811cell after 10 cycles, and c) Li|PEO-oxide SSE|NCM811cell after 50 cycles.


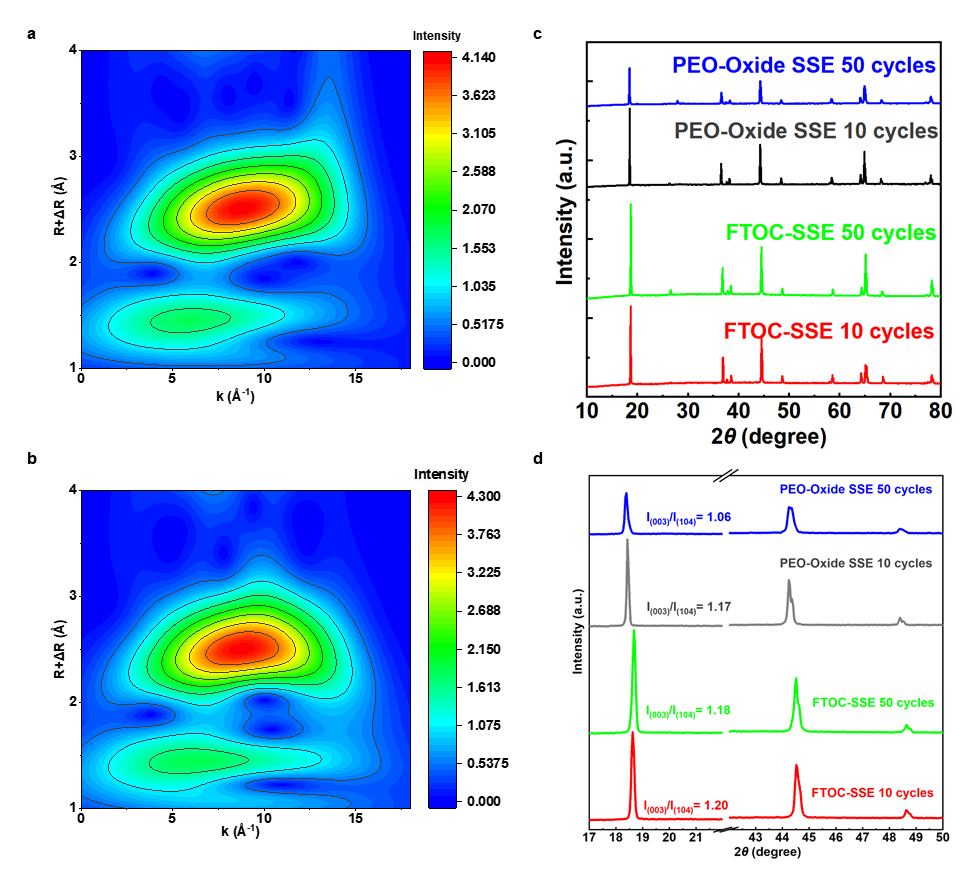


**Figure S39.** 2D XANES results of NCM811 electrodes harvested from a Li|FTOC-SSE|NCM811cell a) after 1 cycle. b) after 10 cycles. c-d) XRD results for NCM811 electrodes harvested from a Li||NCM811cell.

**Table S1:** Crystallinity of different SSEs.

| Electrolyte | ${\Delta H}_{m}^{Sample}$ (J/g） | ${\Delta H}_{m}^{*PEO}$ (J/g) | Crystallinity *χ*_c_ (%) |
| --- | --- | --- | --- |
| PEO-SSE | 34.6 | 203.0 | 17.5 |
| PEO-Oxide SSE | 32.1 | 203.0 | 15.8 |
| FTOC-SSE | 20.6 | 203.0 | 10.1 |

Crystallinity calculation formula, where *χ*_c_ represents the crystallinity and Δ*H* denotes the enthalpy.

$$\chi=\frac{{\Delta H}_{m}^{Sample}}{{\Delta H}_{m}^{*PEO}}⨯100\%$$

**Table S2: Comparative data from recent works of other groups**

| Ref. | Electrolyte,  Full-cell description | Ionic conductivity,  Electrochemical stable window (ESW) | Electrolyte type, Configuration,  Temperature | Group |
| --- | --- | --- | --- | --- |
| ^[12]^ | PEO-based PL-ZCNF,  LFP\|\|Li for 900 cycles, 5C | 5.37×10⁻^4^ S cm⁻^1^ (60°C),  ESW= 4.5 V | EO: Li=10,  150 µm,  60 °C | High Li-salt concentration,  thickness,  single operating temperature |
| ^[13]^ | PEO-based SPC (1,4-DITFB),  LFP\|\|Li for 420 cycles, 0.1C | 1.20×10⁻^4^ S cm⁻^1^ (30°C), | EO: Li=10,  80 µm,  30 °C | High Li-salt concentration,  single operating temperature |
| ^[14]^ | PEO-based CPCSE (GdOOH),  NCM811\|\|Li for 250 cycles, 0.5C | 5.2×10⁻^4^ S cm⁻^1^ (30°C),  ESW= 5.1V | EO: Li=8,  110 µm,  60 °C | High Li-salt concentration,  thickness.  single operating temperature |
| ^[15]^ | PEO-based CPE (Al_2_O_3_+MgClO_4_),  NCM811\|\|Li for 120 cycles, 0.2C | 2.15×10⁻^4^ S cm⁻^1^ (30°C), | EO: Li=10,  60 °C | High Li-salt concentration,  single operating temperature |
| ^[16]^ | PEO+LGPS CPE,  Li_2_O\|\|Li for 1000 cycles | 5.2×10⁻^4^ S cm⁻^1^ (30°C),  ESW~5.27 | EO: Li=10,  RT | High Li-salt concentration,  single operating temperature |
| ^[17]^ | PEO+LATP+PAN DPCE,  NCM622\|\|Li for 120 cycles, 0.5C | 6.26×10⁻^4^ S cm⁻^1^ (60°C),  ESW= 4.5 V | EO: Li<10,  ~340 µm,  60 °C | High Li-salt concentration,  thickness.  single operating temperature |
| ^[18]^ | PVDF+LLZTO+PEO SSE,  LMFP\|\|Li for 100 cycles, 0.2C | 5.73×10⁻^5^ S cm⁻^1^ (40°C) | EO: Li<14,  65 °C | Single operating temperature |
| ^[19]^ | POE+PEO SSE,  TiO_2_@NCM622\|\|Li for 100 cycles,  0.1 mA cm⁻^2^ | 7.1×10⁻^5^ S cm⁻^1^ (65°C),  ESW= 5.0 V | EO: Li<14,  100 µm,  65 °C | Thickness.  single operating temperature |
| ^[20]^ | PEO+PMA SSE,  LCO\|\|Li for 100 cycles, 0.1 mA cm⁻^2^ | 1.39×10⁻^4^ S cm⁻^1^ (65°C), | EO: Li<14,  130 µm,  65 °C | Thickness.  single operating temperature |
| ^[21]^ | PEO-C4P SSE,  LFP\|\|Li for 1000 cycles, 1C | 1.9×10⁻^3^ S cm⁻^1^ (60°C), | EO: Li=16,  60 °C | Single operating temperature |
| ^[22]^ | PEO-AOC SSE,  LFP\|\|Li for 200 cycles, 0.25 mA cm⁻^2^ | 11.8×10⁻^4^ S cm⁻^1^ (35°C) | EO: Li=15,  50 °C | Single operating temperature |
| ^[23]^ | PEO-Mg-Al CPE,  Ni83\|\|Li for 300 cycles, 0.9 mAh cm⁻^2^ | 2.3×10⁻^4^ S cm⁻^1^ (RT) | EO:Li=18,  60 °C | Single operating temperature |
| ^[24]^ | PEO-ASPE (CD),  LMFP\|\|Li for 200 cycles, 42 mA g⁻^1^ | 6.43×10⁻^4^ S cm⁻^1^ (80°C)  ESW=4.7 V | 200 µm,  70 °C | Thickness.  single operating temperature |
| ^[25]^ | PEO+AlF_3_ CPE,  LFP\|\|Li for 1200 cycles, 50 mA g⁻^1^ | 4.63×10⁻^5^ S cm⁻^1^ (30°C) | 200 µm,  60 °C | Thickness.  single operating temperature |
| ^[26]^ | HEMI-ASPE,  LFP\|\|Li for 300 cycles, 70 mA g⁻^1^ | 2.17×10⁻^4^ S cm⁻^1^ (70°C), | 100 µm,  70 °C | Thickness.  single operating temperature |
|  | **NCM811\|\|Li**  **400 cycles,**  **LMFP\|\|Li for 1200 cycles** | **2.10×10⁻^4^ S cm⁻^1^ (30°C),** | **EO:Li=16,**  **20~40 µm,**  **30~100 °C** | **This work** |

**Summary of recent studies on PEO-based electrolytes.** High lithium salt concentrations and increased electrolyte thickness have been shown to improve the cycle life of solid-state batteries, particularly under high-voltage conditions. However, the introduction of foreign metal ions and the presence of highly reactive species further exacerbate the challenges associated with ensuring stable operation at elevated temperatures.

**References**

[1] C. Zhao, Y. Han, S. Dai, X. Chen, J. Yan, W. Zhang, H. Su, S. Lin, Z. Tang, B. K. Teo, N. Zheng, *Angew. Chem. Int. Ed.* **2017**, *56*, 16252.

[2] B. Ravel, M. Newville, *J. Synchrotron Radiat.* **2005**, *12*, 537.

[3] Frisch, M., Trucks, G., Schlegel, H., Scuseria, G., Robb, M., Cheeseman, J., et al., **2016**. Gaussian 16; Gaussian, Inc: Wallingford, CT.

[4] Zhao, Y., Truhlar, D.G., **2008**. The M06 suite of density functionals for main group thermochemistry, thermochemical kinetics, noncovalent interactions, excited states, and transition elements: two new functionals and systematic testing of four M06-class functionals and 12 other functionals. Theor. Chem. Acc. 120, 215-241.

[5] Zhang J, Zhang W, Wang Y, Jiang S, Wang Y, Liu X, et al. Degradation of methyl parathion in thermally activated peroxymonosulfate processes: Kinetics, reaction mechanism and toxicity evaluation. J. Hazard. Mater., **2025**, 491, 137987.

[6] C. He, H. Ying, L. Cai, H. Chen, Z. Xu, S. Liu, P. Huang, H. Zhang, W. Song, J. Zhang, L. Shi, W. Gao, D. Li, W. Han, *Adv. Funct. Mater.* **2024**, 2410350.

[7] R. Li, H. Hua, X. Yang, J. Tian, Q. Chen, R. Huang, X. Li, P. Zhang, J. Zhao, *Energy Environ. Sci.* **2024**, *17*, 5601.

[8] Y. Fan, O. I. Malyi, H. Wang, X. Cheng, X. Fu, J. Wang, H. Ke, H. Xia, Y. Shen, Z. Bai, S. Chen, H. Shao, X. Chen, Y. Tang, X. Bao, *Angew. Chem.* **2025**, *137*, e202421777.

[9] T. Wang, B. Chen, C. Liu, T. Li, X. Liu, *Angew. Chem. Int. Ed.* **2024**, *63*, e202400960.

[10] J. Park, J. Jeong, Y. Lee, M. Oh, M. Ryou, Y. M. Lee, *Adv. Mater. Interfaces* **2016**, *3*, 1600140.

[11] W. Wang, Y. Yang, J. Yang, J. Zhang, *Angew. Chem. Int. Ed.* **2024**, *63*, e202400091.

[12] Y. Cheng, Z. Cai, J. Xu, Z. Sun, X. Wu, J. Han, Y. Wang, M. Wang, *Angew. Chem. Int. Ed.* **2024**, *63*, e202400477.

[13] H. Zhou, Y. Ou, S. Yan, J. Xie, P. Zhou, L. Wan, Z. Xu, F. Liu, W. Zhang, Y. Xia, K. Liu, *Angew. Chem. Int. Ed.* **2023**, *62*, e202306948.

[14] Y. Cheng, X. Liu, Y. Guo, G. Dong, X. Hu, H. Zhang, X. Xiao, Q. Liu, L. Xu, L. Mai, *Adv. Mater.* **2023**, *35*, 2303226.

[15] B. Xu, X. Li, C. Yang, Y. Li, N. S. Grundish, P.-H. Chien, K. Dong, I. Manke, R. Fang, N. Wu, H. Xu, A. Dolocan, J. B. Goodenough, *J. Am. Chem. Soc.* **2021**, *143*, 6542.

[16] A. Kondori, M. Esmaeilirad, A. M. Harzandi, R. Amine, M. T. Saray, L. Yu, T. Liu, J. Wen, N. Shan, H.-H. Wang, A. T. Ngo, P. C. Redfern, C. S. Johnson, K. Amine, R. Shahbazian-Yassar, L. A. Curtiss, M. Asadi, *Science* **2023**, *379*, 499.

[17] J.-Y. Liang, X.-X. Zeng, X.-D. Zhang, T.-T. Zuo, M. Yan, Y.-X. Yin, J.-L. Shi, X.-W. Wu, Y.-G. Guo, L.-J. Wan, *J. Am. Chem. Soc.* **2019**, *141*, 9165.

[18] J. Zhu, S. He, H. Tian, Y. Hu, C. Xin, X. Xie, L. Zhang, J. Gao, S. Hao, W. Zhou, L. Zhang, *Adv. Funct. Mater.* **2023**, *33*, 2301165.

[19] X. Pan, H. Sun, Z. Wang, H. Huang, Q. Chang, J. Li, J. Gao, S. Wang, H. Xu, Y. Li, W. Zhou, *Adv. Energy Mater.* **2020**, *10*, 2002416.

[20] W. Zhou, Z. Wang, Y. Pu, Y. Li, S. Xin, X. Li, J. Chen, J. B. Goodenough, *Adv. Mater.* **2019**, *31*, 1805574.

[21] J. Tian, J. Ji, Y. Zhu, Y. He, H. Li, Y. Li, D. Luo, J. Xing, L. Qie, J. L. Sessler, X. Chi, *Adv. Mater.* **2024**, *36*, 2308507.

[22] C.-D. Fang, Y. Huang, Y.-F. Sun, P.-F. Sun, K. Li, S.-Y. Yao, M.-Y. Zhang, W.-H. Fang, J.-J. Chen, *Nat. Commun.* **2024**, *15*, 6781.

[23] H. An, M. Li, Q. Liu, Y. Song, J. Liu, Z. Yu, X. Liu, B. Deng, J. Wang, *Nat. Commun.* **2024**, *15*, 9150.

[24] Y. Su, X. Rong, A. Gao, Y. Liu, J. Li, M. Mao, X. Qi, G. Chai, Q. Zhang, L. Suo, L. Gu, H. Li, X. Huang, L. Chen, B. Liu, Y.-S. Hu, *Nat. Commun.* **2022**, *13*, 4181.

[25] J. Hu, C. Lai, K. Chen, Q. Wu, Y. Gu, C. Wu, C. Li, *Nat. Commun.* **2022**, *13*, 7914.

[26] Y. Su, X. Rong, H. Li, X. Huang, L. Chen, B. Liu, Y. Hu, *Adv. Mater.* **2023**, *35*, 2209402.
